# Trace metals from historical mining sites and past metallurgical activity remain bioavailable to wildlife today

**DOI:** 10.1038/s41598-018-20983-0

**Published:** 2018-02-21

**Authors:** Estelle Camizuli, Renaud Scheifler, Stéphane Garnier, Fabrice Monna, Rémi Losno, Claude Gourault, Gilles Hamm, Caroline Lachiche, Guillaume Delivet, Carmela Chateau, Paul Alibert

**Affiliations:** 1 UMR 6298, ArTeHiS, Université Bourgogne Franche-Comté–CNRS, Dijon, 21000 France; 2 UMR 5204 EDYTEM, Université Savoie Mont Blanc–CNRS, Le Bourget-du-Lac cedex, 73376 France; 3 UMR 6249, Chrono-Environnement, Université Bourgogne Franche-Comté–CNRS, Besançon, 25000 France; 4 UMR 6282, Biogéosciences, Université Bourgogne Franche-Comté–CNRS, Dijon, 21000 France; 5 IPGP, Paris, cedex 05 75238 France; 6 UFR SVTE, Université Bourgogne Franche-Comté, 21000 Dijon, France

## Abstract

Throughout history, ancient human societies exploited mineral resources all over the world, even in areas that are now protected and considered to be relatively pristine. Here, we show that past mining still has an impact on wildlife in some French protected areas. We measured cadmium, copper, lead, and zinc concentrations in topsoils and wood mouse kidneys from sites located in the Cévennes and the Morvan. The maximum levels of metals in these topsoils are one or two orders of magnitude greater than their commonly reported mean values in European topsoils. The transfer to biota was effective, as the lead concentration (and to a lesser extent, cadmium) in wood mouse kidneys increased with soil concentration, unlike copper and zinc, providing direct evidence that lead emitted in the environment several centuries ago is still bioavailable to free-ranging mammals. The negative correlation between kidney lead concentration and animal body condition suggests that historical mining activity may continue to play a role in the complex relationships between trace metal pollution and body indices. Ancient mining sites could therefore be used to assess the long-term fate of trace metals in soils and the subsequent risks to human health and the environment.

## Introduction

The first evidence of extractive metallurgy dates from the 6^th^ millennium BC in the Near East^1,2^. Since then, mining and smelting activities have developed almost everywhere that humans have settled^3,4^, resulting in the emission of unexpectedly large amounts of metals into the environment, e.g., during the Roman Empire^5,6^. Deleterious consequences on human health were observed as early as the 1^st^ century BC, with Lucretius, for instance, pointing out *“the ill effects in the miners’ complexions”* and writing *“How deadly are the exhalations of gold mines!”* (*De natura rerum*, 4, 808^7^). Negative impacts of mining and smelting activities on animals and the environment were also recognized long ago. During the 1^st^ century BC, Vitruvius wrote that springs coming from mining areas were very harmful (*De Architectura*, 8, 5^8^), while Pliny the Elder, during the 1^st^ century AD, noticed how silver mine emissions affect all animals (*Naturalis Historia*, 33, 31^9^).

With geographical shifts of human settlements over time, some mining and/or smelting sites may have vanished from collective memory^10–12^. For instance, in the Morvan and Cévennes massifs (France), the older sites remain difficult to identify in the field, particularly in forested areas. Because of their outstanding landscapes and biodiversity, both the Morvan and the Cévennes are recognized as nature parks, considered to be pristine areas, relatively free from anthropogenic impact. These areas have nonetheless experienced several phases of mining and smelting, starting as early as the Bronze Age for the Morvan^13–15^ and at least from the Iron Age for the Cévennes^16^.

In these parks, recent archaeological studies have identified ancient metallurgical sites, their spatial extent and the nature of their activities, together with palaeoenvironmental information^17,18^. Companion geochemical studies on those sites have shown that soils and sediments can still be highly contaminated by various metals due to their persistence in the environment^10,19^. However, a high concentration of metals in the abiotic environment does not necessarily imply that any transfer to biota will be sufficient to cause adverse effects on organisms^20,21^. The transfer of an element from abiotic compartments to biota depends on the biological characteristics of the targeted organisms as well as the bioavailability of the element, which is influenced by the physico-chemical properties of both the pollutant and the medium^22^. At sites that have been contaminated in the past but are no longer subject to polluting activities, bioavailability may have drastically decreased because of various physico-chemical processes that immobilise metals in abiotic compartments, e.g., soils^23^. The degree of toxicity, once a metal has been transferred into an organism, depends on the type of metal and on the defence mechanisms deployed by the organism (excretion, storage under non- or less toxic chemical forms of the metal, etc.). Metals can be classified into two categories, according to their physiological role: “essential” elements are those metals that have a crucial biological function in organisms (such as iron in haem, a component of haemoglobin), while “non-essential” elements are those for which no biological function is known. Any deleterious effects of non-essential elements generally occur at lower relative concentrations than those of essential elements, which can, however, still be toxic at high levels.

**Figure 1:**
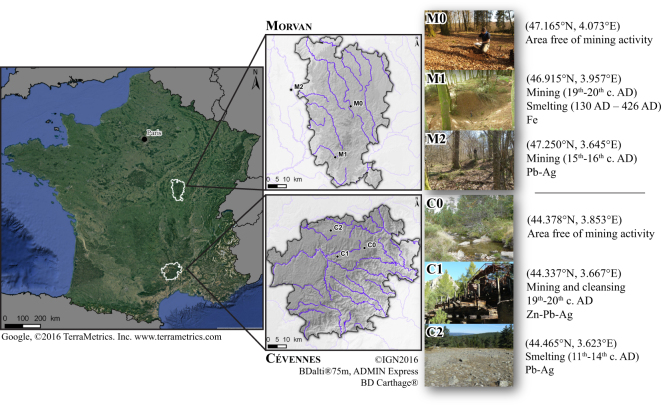
Location of the study sites where soils and small mammals were sampled. The Morvan Regional Nature Park and the Cévennes National Park are both located in the Massif Central, France. Within each park, three sites were selected based on their degree of contamination. M0 in the Morvan and C0 in the Cévennes are free of mining and were used as reference sites. The coordinates of the centroid are given in WGS84 (EPSG 4326), decimal degrees (Lat, Long). The maps were created using QGIS software (QGIS Essen 2.14.6, http://www.qgis.org), adapted by E. Camizuli from Google Satellite©2016 and IGN©2016.

We therefore investigated whether trace metals (TMs) in soils surrounding ancient mining and metallurgical sites from various periods in two parks, the Morvan Regional Nature Park and the Cévennes National Park, are still bioavailable and, if so, toxic to wildlife. The aims of the present study were (*i*) to quantify the level of soil contamination by four TMs directly linked to mining activity, (*ii*) to check whether these contaminants were bioavailable to organisms such as the wood mouse, and finally (*iii*) to see if contamination ever occurred at levels prejudicial to the organism’s health. Within each park, three sites were selected (Fig. 1): one free of mining, used as a reference site (M 0 in the Morvan and C 0 in the Cévennes), one moderately contaminated (M1 in the Morvan and C1 in the Cévennes), and one highly contaminated (M2 in the Morvan and C2 in the Cévennes). Four TMs (two essential elements, copper (Cu) and zinc (Zn), and two non-essential elements, cadmium (Cd) and lead (Pb)), were measured in topsoils (*n* = 261) and in the kidneys of wood mice (*Apodemus sylvaticus*, *n* = 157) sampled at the six study sites. These four elements were selected because of the local geology of the study sites, where past mining activities mainly exploited polymetallic sulphide ores. The potential toxic effects of these elements on the local fauna were investigated by several proxies: body condition for nutritional status^24–27^, somatic indices for possible histological damage^28^, and fluctuating asymmetry (FA) for developmental instability^29,30^.

## Results

### Trace metal concentrations in topsoils

**Figure 2:**
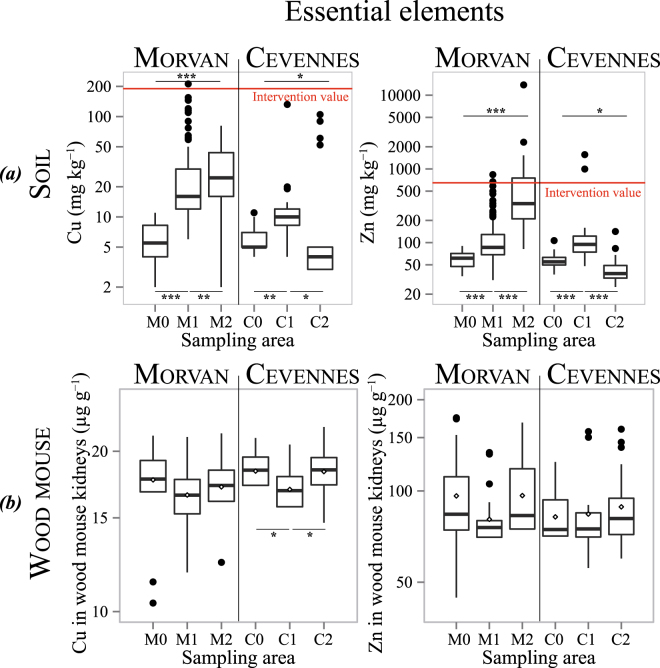
(**a**) Distribution of topsoil concentrations of essential elements (Cu and Zn) at the six study sites. Dutch intervention values for assessing soil contamination are represented by a red line^31^ (**b**) Distribution of essential elements (Cu and Zn) kidney concentrations in wood mice sampled at the six study sites (dry mass basis in). 0 < *** < 0.001 < ** < 0.01 < * < 0.05 < . < 0.1.

**Figure 3:**
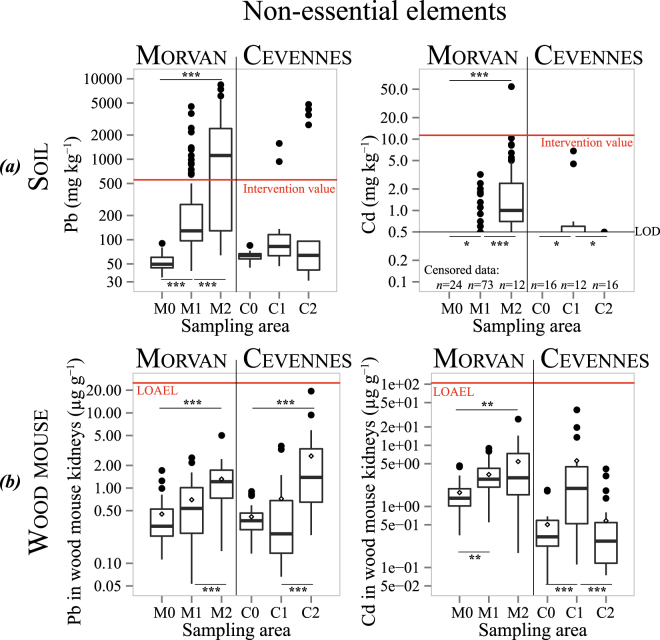
(**a**) Distribution of topsoil concentrations of non-essential elements (Pb and Cd) at the six study sites. Dutch intervention values for assessing soil contamination are represented by a red line^31^ (**b**) Distribution of non-essential elements (Pb and Cd) kidney concentrations in wood mice sampled at the six study sites (dry mass basis in). The Lowest Observed Adverse Effect Levels (LOAELs) defined by Shore & Douben^43,44^ are represented by a red line. 0 < *** < 0.001 < ** < 0.01 < * < 0.05 < . < 0.1.

The TM concentrations in soils ranged from less than 0.5 mg·kg^−1^ to 54.2 mg·kg^−1^ for Cd, 11 mg·kg^−1^ to 212 mg·kg^−1^ for Cu, 85 mg·kg^−1^ to 8410 mg·kg^−1^ for Pb, and 90 mg·kg^−1^ to 13800 mg·kg^−1^ for Zn (Table 1 and Supplementary Table S1). Maximum levels for all the four TMs studied were found in the contaminated sites of the Morvan region. The spatial distribution of TMs in soils shows that higher concentrations were found in the mining and metallurgical sites, whatever the region considered (Fig. 2a, Fig. 3a, Supplementary Fig. S1 for the Morvan and Supplementary Fig. S2 for the Cévennes). In the Morvan, the three sites differed significantly (Kruskal-Wallis test, all *p* < 0.05) in terms of Cd, Cu, Pb and Zn contents in topsoils (Fig. 2a and Fig. 3a), with all elements following the pattern *M*0 < *M*1 < *M*2 (Steel-Dwass pairwise comparisons). In the Cévennes, the three sites differed for Cu and Zn contents with the pattern *C*2 < *C*0 < *C*1 (Fig. 2a). The Cd concentrations were similar in C0 and C2, both were lower than in C1. The Pb concentrations were similar for the three study sites (Kruskal-Wallis test, *p* > 0.05) but C2 exhibited greater spatial heterogeneity, ranging from 31 mg·kg^−1^ to 4810 mg·kg^−1^ (Fig. 3a and Supplementary Table S1).

**Table 1:** Maximum concentrations for Cd, Cu, Pb and Zn in topsoils of the six study sites compared to reference values.

		Cd (mg · kg^−1^)	Cu (mg · kg^−1^)	Pb (mg · kg^−1^)	Zn (mg · kg^−1^)
Morvan	M0	<0.5 (LOD)	11	90	90
	M1	3.2	212	4520	835
	M2	54.2	81	8410	13800
Cévennes	C0	<0.5 (LOD)	11	85	107
	C1	6.8	132	1580	1560
	C2	10	105	4810	142
European Topsoils^32^	Mean value	0.28	17.3	32	68.1
French sewage sludge for amendment*	Content limit	2	100	100	300
Dutch standards^31^	Intervention value	12	190	530	720

*Under French regulations, sewage sludge for agricultural soil amendment must not contain trace metal concentrations above these limits. LOD stands for Limit Of Detection.

### Wood mouse population characteristics

The wood mouse age structures differed significantly between the two parks (*χ*^2^ = 8.48, *p* = 0.01, *df* = 2), but not between the three sites for either the Morvan (*χ*^2^ = 1.85, *p* = 0.76, *df* = 4) or the Cévennes (*χ*^2^ = 5.00, *p* = 0.29, *df* = 4). The sex ratios did not differ significantly between parks or sites (*χ*^2^ = 0.47, *p* = 0.49, *df* = 1 for parks, *χ*^2^ = 2.22, *p* = 0.33, *df* = 2 for the Morvan and *χ*^2^ = 1.01, *p* = 0.6, *df* = 2 for the Cévennes, Supplementary Fig. S3).

### Trace metal concentrations in kidneys

The Cu concentrations in wood mouse kidneys ranged from 10.37 μg·g^−1^ to 22 μg·g^−1^, and the Zn concentrations ranged from 44.5 μg·g^−1^ to 160 μg·g^−1^ (Supplementary Table S2). Except for Cu in the Cévennes with *C*1 < *C*0 − *C*2 (Tukey’s HSD test after an analysis of variance (ANOVA) with *p* < 0.05), essential element concentrations did not differ between the study sites (*p* > 0.05), suggesting physiological (homeostatic) regulation (Fig. 2b). Concentrations of the non-essential elements in the kidneys ranged from 0.05 μg·g^−1^ to 38 μg·g^−1^ for Cd and from 0.05 μg·g^−1^ to 19 μg·g^−1^ for Pb. Maximum concentrations were found in the contaminated sites of the Cévennes region (*C*1 for the Cd and *C*2 for Pb). The Cd concentrations followed these patterns: *M*0 < *M*1 − *M*2 and *C*0 − *C*2 < *C*1. The Pb concentrations followed the same pattern in both parks, showing no differences between sites 0 and 1, but with both values lower than for site 2 (Fig. 3b).

### Trace metal concentrations in wood mice in relation to biological and environmental parameters

**Figure 4:**
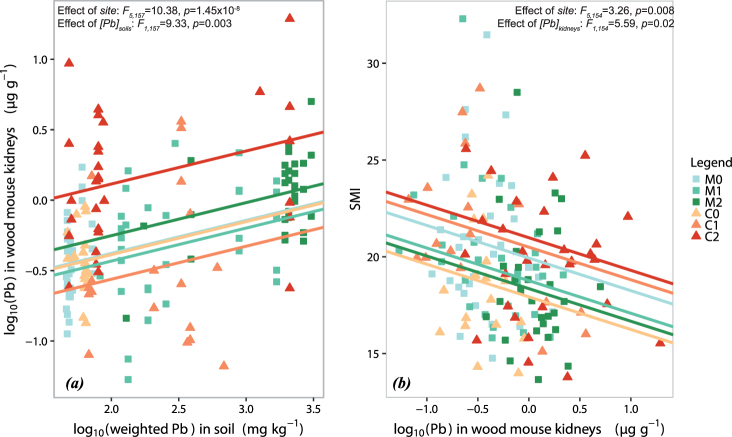
The effect of Pb concentrations on wood mice. (**a**) Variation of Pb concentrations in wood mouse kidneys in relation to both Pb concentrations in soils (abscissa) and sites (illustrated by different colours). (**b**) Variation of body condition as assessed by scaled mass index (SMI) in relation to both Pb concentrations in wood mouse kidneys (abscissa) and sites (illustrated by different colours). As the model was complex, we present only two parameters influencing the SMI (Pb concentrations in wood mice and site).

Multivariate linear models were used to investigate the relationship between TM concentrations in kidneys and explanatory variables (site, TMs in soils, sex, and mass), for each TM separately. Both Cd and Zn concentrations in wood mouse kidneys were best explained by models combining study sites and body mass (Table 2, for description of best-fit models and Supplementary Table S3 for model parameters). The same pattern was observed for Cu concentrations in wood mouse kidneys, with sex as an additional factor (Table 2 and Supplementary Table S3). The Pb concentrations varied between sites as indicated above and increased with Pb concentrations in soil (Table 2, Fig. 4a and Supplementary Table S3). The Cd concentrations in wood mouse kidneys increased slightly with mass, while Cu and Zn concentrations decreased slightly (Table 2 and Supplementary Table S3).

**Table 2:** Summary of best-fit models for trace metals in wood mouse kidneys.

Best-fit models	*n*	*Df*	*F* value	*Pr*(>*F*)	*R* ^2^
${\mathrm{log}}_{10}(C{u}_{{kidneys}})\sim {site}+{sex}+{mass}$	**157**				**0.60**
*site*		5	2.50	0.03*	
*sex*		1	5.24	0.02*	
*mass*		1	21.03	9.51 ⋅ 10^−06^***	
${\mathrm{log}}_{10}(Z{n}_{{kidneys}})\sim {site}+{mass}$	**157**				**0.14**
*site*		5	2.56	0.03*	
*mass*		1	13.93	0.0003***	
${\mathrm{log}}_{10}(P{b}_{{kidneys}})\sim site+{\mathrm{log}}_{10}(P{b}_{soil})$	**157**				**0.35**
*site*		5	10.38	1.45 ⋅ 10^−08^***	
${\mathrm{log}}_{10}(P{b}_{soil})$		1	9.33	0.003**	
${\mathrm{log}}_{10}(C{d}_{kidneys})\sim site+mass$	**79**				**0.23**
*site*		2	4.25	0.02*	
*mass*		1	18.92	4.24 ⋅ 10^−05^***	

Models relating trace metal concentrations in kidneys to biological and environmental parameters, and trace metal concentrations in soils. 0 < *** < 0.001 < ** < 0.01 < * < 0.05 < . < 0.1.

### Toxic effects assessed by body condition, somatic indices, and fluctuating asymmetry

Relationships between body condition and somatic indices were investigated with multivariate linear models using study sites, the four TMs in wood mouse kidneys, and sex as explanatory variables. Body condition as assessed by scaled mass index (SMI) was best explained by a model combining study sites, Cd and Pb concentrations in kidneys and the interaction between Cu or Zn concentrations in kidneys and sex (Table 3). The SMIs varied according to the sites, increased with Cd concentrations and were negatively related to Pb concentrations in kidneys (Fig. 4b and Supplementary Table S4). The SMIs were negatively influenced by the interaction between Cu concentrations and sex, and positively influenced by the interaction between Zn concentrations and sex. Somatic index data (scaled liver index - SLI, and scaled kidneys index - SKI) were best fit by models including study sites, sex and the interaction between Cu or Zn concentrations in kidneys and sex (Table 3). Like SMI, both SKI and SLI were negatively related to the Cu × sex interaction and positively related to the Zn × sex interaction (Supplementary Table S4). Concerning FA, preliminary tests were performed for all traits measured (here, length and width of lower molars) as recommended by Palmer^29^. These tests did not suggest that directional asymmetry (DA), antisymmetry (AS) or relationships between asymmetry and trait size could significantly bias FA estimates (Supplementary Table S5). No significant correlations were found between absolute asymmetry distribution and TM concentrations in kidneys (Supplementary Table S6). When compared to measurement error, FA10, a parameter used for FA assessment, was always significant (Supplementary Table S7), but no clear relationship was found between levels of developmental instability assessed by FA and sites for either park (Supplementary Fig. S4).

**Table 3:** Summary of best-fit models for body condition and somatic indices.

Best-fit models	*n*	*Df*	*F* value	*Pr*(>*F*)	*R* ^2^
$$SMI\sim site+{\mathrm{log}}_{10}Cd+{\mathrm{log}}_{10}Cu+{\mathrm{log}}_{10}Pb+{\mathrm{log}}_{10}Zn+sex+{\mathrm{log}}_{10}Cu\times sex+{\mathrm{log}}_{10}Zn\times sex$$					
	**154**				**0.24**
*site*		5	3.26	0.008**	
${log}_{10}(C{d}_{kidneys})$		1	4.58	0.03*	
${log}_{10}(P{b}_{kidneys})$		1	5.59	0.02*	
${log}_{10}(C{u}_{kidneys})\times sex$		1	10.78	0.001**	
${log}_{10}(Z{n}_{kidneys})\times sex$		1	11.45	0.0009***	
$$SLI\sim site+{\mathrm{log}}_{10}Cu+{\mathrm{log}}_{10}Zn+sex+{\mathrm{log}}_{10}Cu\times sex+{\mathrm{log}}_{10}Zn\times sex$$					
	**155**				**0.30**
*site*		5	7.28	4.26 ⋅ 10^−06^***	
${log}_{10}(C{u}_{kidneys})\times sex$		1	10.05	0.002**	
${log}_{10}(Z{n}_{kidneys})\times sex$		1	5.88	0.02*	
$$SKI\sim site+{\mathrm{log}}_{10}Cu+{\mathrm{log}}_{10}Zn+sex+{\mathrm{log}}_{10}Cu\times sex+{\mathrm{log}}_{10}Zn\times sex$$					
	**155**				**0.25**
*site*		5	5.38	0.0001***	
${log}_{10}(C{u}_{kidneys})\times sex$		1	10.95	0.001**	
${log}_{10}(Z{n}_{kidneys})\times sex$		1	10.52	0.001**	

Models relating body condition and somatic indices to biological and environmental parameters and trace metal concentrations in wood mouse kidneys. 0 < *** < 0.001 < ** < 0.01 < * < 0.05 < . < 0.1.

## Discussion

Even centuries after mining and metallurgical activities have ceased, TM concentrations in soils surrounding such sites still reach high levels. There is no European consensus on threshold concentrations of metals in soils, but the Dutch government proposed a soil classification scheme in which intervention values are defined. These strict values identify serious contamination of soils (12, 190, 530, and 720 mg·kg^−1^ dry matter for Cd, Cu, Pb, and Zn, respectively) and indicate that remediation is necessary^31^. In the present study, 75 out of 261 soil samples (29%) exceed the Pb intervention value, reaching 59% in the highly contaminated Morvan site, M2 (Fig. 3a). For both Cd and Cu, only one sample out of 261 exceeds the Dutch intervention values, while Zn exhibits an intermediate pattern, with 10% of the soils exceeding the threshold (Fig. 2a). Among the four metals studied and according to this classification, Pb concentrations represent the most important risk, which is probably linked to the nature of the ore exploited (galena) and to the low mobility of Pb in the environment^32^. For Cd, while only one soil exceeds the Dutch intervention threshold, this intervention limit is high (12 mg·kg^−1^) compared to the average concentration in European surface soils (0.28 mg·kg^−1^, Table 1)^32^. In France, Cd concentrations in sewage sludge-amended agricultural soils must be lower than 2 mg·kg^−1^ (Table 1). As Cd is toxic to organisms at low doses, it should also be considered for risk assessment. Maximum levels of Cd and Zn were around 200 times higher than their commonly reported values in European topsoils, while Cu was 12 times and Pb 2–3 times higher^32^ (Table 1). Apart from these locally high concentrations, TM contents in topsoils are highly heterogeneous (Supplementary Fig. S1 and Fig. S2), complicating risk assessment. Such heterogeneity is also observed in modern mining or smelting sites^33,34^ and can be explained by the spatial distribution of exploitation facilities in relation to the surrounding habitat (e.g., interception of metals emitted in the air by the canopy^35^).

Metal toxicity depends not only on the concentration of a substance in a medium but also on its bioavailability, a complex combination of the physico-chemical characteristics of the pollutant in abiotic compartments and the biological characteristics of organisms^36^. The first component, known as “environmental availability”, represents the physico-chemically driven desorption processes that determine the mobile proportion of the total metal concentration in a soil. Among the numerous parameters that determine this availability, time generally lead to immobilisation of metals, a process named “ageing” ^22,23^. The second component, known as “environmental bioavailability”, represents physiologically driven uptake processes that occur when an organism and a pollutant co-occur in time and space (namely, the “exposure” of an organism). In the present work, our aim was to examine whether these high, spatially heterogeneous concentrations of metals were bioavailable to organisms by measuring TM levels in wood mouse kidneys. For the two essential elements, concentrations did not differ among sites, suggesting their efficient (homeostatic) regulation^37^. Concentrations measured here are similar to values found in other studies on wood mice^38,39^. However, the non-essential elements Cd and Pb showed marked differences between sites, with higher concentrations in the mining and smelting areas, showing that these metals are still bioavailable to wildlife. Concentrations of Pb in wood mouse kidneys measured in the present study are of the same order of magnitude as those measured in animals sampled around a non-ferrous smelter still in activity in Antwerp (Belgium)^38^. They are, however, below the concentrations observed in wood mice in the surroundings of Metaleurop Nord, a Pb and Zn smelter in activity from 1894 to 2003 in northern France^28,40^. Direct comparisons between all these values must be undertaken with caution because the sites differ in terms of metal concentrations in soils, soil properties, and/or exposure (diet). Biota-to-soil accumulation factors (BSAFs)^41^, i.e. the ratio of TM concentrations in organisms to TM concentrations in soils, can be used to compare TM transfer. In the present study, the BSAF for Pb at the most contaminated Morvan site (*M*2, median Pb concentration in soils of 1115) is 0.0008, approximately three times lower than the value measured (0.0029) at a Metaleurop Nord site, which presented a similar Pb concentration in soils (median Pb concentration in soils of 1357)^40^. This lower value suggests less Pb transfer in the present study, which may indicate lower availability of this metal in soils affected by ancient mining contamination, as suggested by Camizuli *et al*.^42^.

Once a pollutant has been taken up by an organism, toxic effects will occur only if various defence mechanisms are overcome. In this study, Cd and Pb concentrations in wood mouse kidneys are below the Lowest Observed Adverse Effect Levels (LOAELs), as defined by Shore and Douben^43,44^ (Fig. 3b), suggesting that toxic effects are unlikely to occur. However, we found a significant negative relationship between SMI and kidney Pb concentrations. The SMI is a measure of body condition that is often defined as a measure of the energetic (or nutritional) state of an animal^25^. Even if the calculation and interpretation of such indices are still much debated^24,27^, these indices are assumed to be related to fitness. Here, we used the SMI, which has recently been shown to be a better indicator of the relative size of energy reserves than condition indices based on ordinary least squares residuals (see Peig and Green^25^, for details). Although several studies have shown a decline in the body condition of small mammals from polluted sites compared to controls^45,46^, confounding factors like food availability, habitat quality or other chemical elements may contribute to the complex relationships that are observed between pollution and body indices^28^. Therefore the negative relationship between SMI and Pb concentrations, observed in this study, cannot simply be interpreted as implying a direct causal relationship. Other relationships that remain complex to interpret are the positive correlation between SMI and Cd concentrations in kidneys, and the interactions between essential element concentrations (Cu and Zn) and sex. These complex relationships between the body condition of free-ranging vertebrates and both essential and non-essential elements clearly require further investigation. Somatic indices (the relative size of internal organs), which may reveal oedema in individuals exposed to toxic compounds^46^, did not exhibit any clear relationship with metals. Concerning FA, relationships between the degree of developmental instability of populations and the level of the environmental and/or genetic stress to which they were subjected have already been demonstrated^10,47^. In this study, FA10 was detected, but no relationship was found with the sampling site. This result is not in agreement with a study on aquatic ecosystems in the Cévennes National Park, which showed that wild trout were affected by increasing developmental instability in relation to mining contamination^10^.

Taken together these results show that several centuries after mining and smelting activities have ceased, metals are still bioavailable to wildlife, with Pb, and to a lesser extent Cd, concentrations increasing in wood mouse kidneys in relation to soil concentrations. Further studies should be undertaken to determine the precise TM transfer mechanisms that occur in our study sites from the environment to animals (ingestion of soil, animal and plant materials, inhalation of contaminated dust/soil particles, and/or direct transfer through dermal contact). The BSAFs suggest, however, that bioavailability might be lower in soils affected by ancient mining than in soils that have been more recently contaminated. Higher concentrations of Pb in the kidneys of individuals from the most contaminated sites and the negative relationship between these concentrations and SMI raise the issue of the present-day consequences of past anthropogenic activities on wildlife. Specific biomarkers of exposure such as the induction of metallothioneins, a protein involved in the homeostasis of essential elements and in the regulation of non-essential ones^40^, could be envisaged in future studies. Biomarkers of toxic effects, for instance related to the oxidative stress that exposure to trace metals may generate^48^ or to histological pathologies^49^, would also provide further insight into the possible toxic effects that may occur in wildlife. Nature Parks are now protected areas and are considered to be relatively pristine, but in the past, they often were the setting for economic and industrial activities. Ancient industrial activities might sometimes have vanished from collective memory but may still represent a risk that deserves investigation.

## Methods

### Topsoil sampling and analysis

Sampling units for topsoils were either 100 m × 100 m (M1, M2, C1) or 200 m × 200 m (M0, C0, C2) plots, because of logistical and time constraints (see Supplementary Fig. S1 for the Morvan and Supplementary Fig. S2 for the Cévennes). Vegetation and litter were removed before topsoil sampling. For each plot, a composite sample of ∼1 kg, stored in a hermetic polyethylene bag, was prepared from 5 auger samples (0–20 cm depth), following a 20 m cross-shaped pattern. In the laboratory, samples were air-dried, sieved to 2 mm, and carefully quartered. Subsamples of the 261 topsoils were finely ground in an acid-cleaned agate mortar for elemental analyses. Concentrations of Cd, Cu, Pb, and Zn were measured by inductively coupled plasma atomic emission spectroscopy (ICP-AES) after pseudo-total *aqua regia* digestion at Actlabs (Ontario, Canada). This chemical method was chosen because we targeted anthropogenic pollution that is easily extractable, and thus bioavailable to wildlife. Analytical quality control was doubly checked (i) by Actlabs measuring 18 replicates, 8 blanks and several certified reference materials (CRMs) and (ii) by inserting another 25 duplicates and JSD-1, JSD-2, BCSS-1 and PACS-1 CRMs (stream, estuarine, and harbour sediments, respectively) as blind samples. The Actlabs protocol set the limits of detection (LODs) at 0.5 mg·kg^−1^ for Cd, 1 mg·kg^−1^ for Cu, 2 mg·kg^−1^ for Pb and 2 mg·kg^−1^ for Zn (see Supplementary Tables S8 and S9 for details).

### Small mammal sampling and analysis

Small mammal sampling was conducted on 10 plots of 100 m × 100 m at each site (Supplementary Figs S1 and S2). These plots were randomly chosen for the two reference sites (M0 and C0) and at the location of anthropogenic activities for the contaminated sites. Wood mice were trapped between mid-September and mid-October 2010. Sampling authorisations were obtained from the DREAL Bourgogne (French regional territory agency in charge) and from the Cévennes National Park. For each of the 10 selected plots, a line of 25 baited traps was set with alternating INRA (door-) and snap-traps, spaced 4 m apart. An extra chamber was added to the classical INRA trap to increase the survival of trapped animals. Traps were set for 3 to 5 consecutive days to ensure an adequate number of samples and were checked and rebaited each morning. The wood mice caught alive were immediately sacrificed by cervical dislocation in accordance with relevant guidelines and regulations^50,51^ and frozen as soon as possible after their capture. They were stored at −20 °C until dissection in the laboratory. All small mammals captured were determined at the species level by molecular analysis (sequencing of the cytochrome b gene) of a tissue sample performed by a service provider (ADNid laboratory). Body wet mass was used as an estimator of age^45,52,53^. Combined with reproductive status, three age categories were constructed: juvenile (J), subadult (SA), and adult (A), as in Peig and Green^26^ (Supplementary Table S10). For all specimens body mass was measured to the nearest 0.01 g and body length to the nearest 0.01 mm. Livers and kidneys were dried to constant mass and weighed to the nearest 0.001 g. Kidneys were finely ground in an acid-cleaned agate mortar. Concentrations of Cd, Cu, and Zn for the kidney samples (*Apodemus sylvaticus*, *n* = 157) were measured by ICP-AES with ultrasonic nebulisation for Cd, while Pb concentrations were measured by ICP-MS, both after total *aqua regia* digestion. Analytical quality was verified using blanks, CRMs (BCR 185 R, bovine liver; NIST 1547, peach leaves; DOLT-4, dogfish liver; DORM-3, fish protein) and duplicates (see Supplementary Tables S11 and S12 for details).

### Ethics statement

The experiments were performed in 2010, i.e. before the application in France of the DIRECTIVE 2010/63/EU OF THE EUROPEAN PARLIAMENT AND OF THE COUNCIL of 22 September 2010 on the protection of animals used for scientific purposes. This European Directive became applicable in 2013, through the “Décret No. 2013-118 du 1er février 2013 relatif à la protection des animaux utilisés à des fins scientifiques”. Before 2013, the capture of non-protected free-ranging rodent species immediately followed by their sacrifice was not considered to be an experiment on live animals and thus did not require protocol approval by an ethical committee. However, as stated above, care was taken to apply euthanasia protocols appropriate for small rodents in the field, and sampling authorizations were obtained from the appropriate administrative bodies.

### Body condition and somatic indices

Body condition was assessed by the scaled mass index (SMI), and somatic indices were estimated using standard major axis (SMA) regression of ln-mass on ln-length as recommended by Peig and Green^25,26^. As the slope of this regression *b*_*SMA*_ did not differ significantly between sites, it was estimated on the entire dataset excluding pregnant females for SMI (*n* = 154), and on the dataset excluding outliers for scaled somatic indices (*n* = 155). The SMA regression consists of estimating the predicted body mass (SMI) or the predicted organ mass (SLI for the liver, SKI for the kidneys) for each individual *i* when body length is standardised. Calculations for SMI follow the equation:

$$SM{I}_{i}={m}_{i}\times \frac{{L}_{0}^{{b}_{SMA}}}{{L}_{i}}$$

where *m*_*i*_ is the body mass and *L*_*i*_ the body length of the individual *i*, *b*_*SMA*_ is the slope of the regression of ln-body mass on ln-body length and *L*_0_ the arithmetic mean of body length for the population.

The scaled liver index SLI_*i*_ and scaled kidney index SKI_*i*_ values were similarly computed using organ mass instead of body mass. In this study, the *b*_*SMA*_ values were 2.90 (95% confidence interval: 2.62–3.20) for *SMI*, 3.54 (95% confidence interval: 3.10–4.05) for *SLI*, and 2.91 (95% confidence interval: 2.55–3.31) for *SKI*. According to Peig and Green (2009, 2010), the *b*_*SMA*_ value for SMI usually lies between 2.5 and 3.2, which can be used as a guideline to identify reliable estimates of the allometric exponent in mammals^28^.

### Weighted TM concentrations in topsoils

To account for the mobility of the wood mouse (home range of 2500 m²)^54^, a weighted TM concentration of the corresponding topsoil was calculated for each individual. This weighted concentration was calculated as the average of the TM concentration in the topsoil of the 8 plots surrounding the plot where the wood mouse was captured, with the capture plot being weighted twice. These weighted TM concentrations were used in the statistical models.

### Data processing and statistical treatment

The Quantum GIS free software^55^ was used for mapping. Statistical treatment and regression analysis used the smart, lmodel2, stats, and pgirmess packages from the R software^56^.

#### Topsoils

The non-parametric Kruskall-Wallis test was used to assess the differences in topsoil TM concentrations between sites in each park because the residues were not normally distributed. When the Kruskall-Wallis test was significant (*p* < 0.05), pairwise comparisons were made using Steel-Dwass *post-hoc* tests^57^.

#### TM in wood mice

A parametric ANOVA test was used to assess the differences in TM concentrations in wood mouse kidneys between sites in each park. When the ANOVA test was significant (*p* < 0.05), pairwise comparisons were made using Tukey’s HSD test. Linear models were used to investigate the relationships between TM concentrations in kidneys and explanatory variables. Multivariate models were built with biological variables (*mass* and *sex*; *age* and *size* were dropped because of their relation to *mass*), geographical variable (*site*), and corresponding weighted TM concentrations in soils as explanatory variables. Biologically meaningful interactions (soil concentrations × sex and mass × sex) were also taken into account. As the range of TM concentration values in relation to sites did not always overlap, testing the interaction site × concentration in soil was not allowed^58^. Statistical treatments for Cd were only performed with the M1, M2, C1 sites, as M0, C0 and M2 presented too many censored data for Cd in soils. The best-fit model was selected using a backward stepwise regression. The drop1{stats} function combined with an *F* test was used; this method corresponds to a type II ANOVA. For each step, the explanatory variable with the largest *p*-value was deleted until the final step, giving the best-fit model, where all *p*-values were below the *α* = 0.05 level (Supplementary Table S13 for model selection). ANOVA tests were performed on the selected models. Model normality was examined by looking at plots of the standardised residuals versus leverage. Model outputs were satisfactory.

#### Body condition and somatic indices

Backward stepwise regression was also applied to determine whether body condition and somatic indices varied with geography, individual characteristics, or levels of individual contamination. Multivariate models were built with biological variables (only *sex*, as we considered SMIs), a geographical variable (*site*), and the four TM concentrations in kidneys as explanatory variables. Biologically meaningful interactions (kidney TM concentrations × sex) were also taken into account (Supplementary Table S14 for model selection).

#### Fluctuating asymmetry

In this study, six bilateral morphometric traits were selected for fluctuating asymmetry (FA) assessment: length and width of the three lower molars (Supplementary Fig. S5). All measurements were performed twice to control for measurement error. The FA consists of subtle random variations between each side (right, R and left, L) of bilateral traits that are supposed to be perfectly identical. These variations reflect the inability of individuals to correct errors occurring during early development. It has been shown that both genetic and environmental stresses decrease developmental stability^59^. The FA has been proposed as a useful tool to assess individual quality^30^. In fact, three types of biological asymmetry can be distinguished on the basis of the analysis of right minus left (R − L) frequency distribution: directional asymmetry (DA), antisymmetry (AS) and FA. The DA shows a pattern of normal R − L variation distributed about a mean point that is significantly different from zero. The AS shows a pattern of R − L variation distributed about a mean point of zero, but with a frequency distribution departing from normality^30^. The FA shows a normal distribution of R − L values with a mean of zero. Among these three asymmetries, only FA provides an estimation of developmental instability. The presence of DA and AS, together with measurement error, can bias FA estimation. A series of preliminary analyses was performed for each study trait as recommended by Palmer^29^. Individual FA levels were then estimated for each trait using absolute asymmetry. Linear models were computed to assess the relationship between |R − L| values and kidney TM concentrations. Population FA levels were estimated for each trait using FA10, i.e., between-sides variance corrected for measurement error, obtained from the results of linear mixed models with sides (fixed) × individuals (random) (see Palmer^29^, for details). Fisher tests were then performed for each trait studied to explore inter-site differences.

### Data availability

All data generated or analysed during the current study are included in this published article and the Supplementary Information file.

## Electronic supplementary material

Supplementary information
