# Supplementary material for: Trace metals from historical mining sites and past metallurgical activity remain bioavailable to wildlife today
Source: Sci Rep. 2018 Feb 21;8:3436. doi: 10.1038/s41598-018-20983-0 (PMC5821829; doi:10.1038/s41598-018-20983-0)
Supplement: Supplementary file 1 — Supplementary information [file 41598_2018_20983_MOESM1_ESM.pdf]

Trace metals from historical mining sites and past metallurgical activity remain bioavailable to wildlife today

Estelle Camizuli[1,2,+,*], Renaud Scheifler[3,+], Stéphane Garnier[4,+], Fabrice Monna[1,+], Rémi Losno[5], Claude Gourault[1], Gilles Hamm[1], Caroline Lachiche[1], Guillaume Delivet[1], Carmela Chateau[6], and Paul Alibert[4]

[1]UMR 6298, ArTeHiS, Université Bourgogne Franche-Comté – CNRS, 21000 Dijon, France
[2]UMR 5204 EDYTEM, Université Savoie Mont-Blanc – CNRS, 73376 Le Bourget-du-Lac cedex, France
[3]UMR 6249, Chrono-Environnement, Université Bourgogne Franche-Comté – CNRS, 25000, Besançon, France
[4]UMR 6282, Biogéosciences, Université Bourgogne Franche-Comté – CNRS, 21000 Dijon, France
[5]IPGP, 75238 Paris cedex 05, France
[6]UFR SVTE, Université Bourgogne Franche-Comté, 21000 Dijon, France
[*]e.camizuli@wanadoo.fr
[+]these authors contributed equally to this work

Supplementary Information

List of Tables

1 **Trace metal descriptive statistics in topsoils for the six study sites.** . . . 2
2 **Characteristics of wood mice sampled on the six study sites.** . . . 6
3 **Parameters of ANOVA tests for trace metal concentrations in wood mouse kidneys.** . . . 7
4 **Parameters of ANOVA tests for body condition and somatic indices.** . . . 8
5 **Details of preliminary tests performed before treating fluctuating asymmetry for the six wood mouse traits studied.** . . . 9
6 **Summary results for the linear regression of —right-left— versus internal concentration in wood mouse kidneys, for each trait studied.** . . . 10
7 **Results of the two-way mixed model ANOVA (side fixed x individual random) with repeated measurement on each side.** . . . 11
8 **Quality control of soil analysis.** . . . 13
9 **Topsoil results.** . . . 14
10 **Use of body weight to calculate wood mouse age.** . . . 15
11 **Quality control of wood mouse analyses.** . . . 16
12 **Results of TMs in wood mouse kidneys.** . . . 17
13 **R results of backward regression for TMs in wood mouse kidneys.** . . . 18
14 **R results of backward regression for condition indices in wood mouse kidneys.** . . . 24

List of Figures

1 **Spatial distribution of TMs in Morvan topsoils.** The maps were created using QGIS software (QGIS Essen 2.14.6, http://www.qgis.org). Adapted by E. Camizuli. . . . 3
2 **Spatial distribution of TMs in Cévennes topsoils.** The maps were created using QGIS software (QGIS Essen 2.14.6, http://www.qgis.org). Adapted by E. Camizuli. . . . 4
3 **Age ratio and sex ratio of wood mouse populations.** . . . 5
4 **FA10 values for each study site.** . . . 12
5 **Bilateral morphological traits selected to study FA in wood mice (*Apodemus sylvaticus*).** . . . 29

Supplementary Table S 1 – **Trace metal descriptive statistics in topsoils for the six study sites.** Min, Max, Mean, Median are indicated for Cu, Pb, and Zn. For Cd, descriptive statistics were estimated using regression on order statistics (ROS). LOD stands for limit of detection, n.cen for number of censored data (i.e. below LOD), % cen for percentage of censored data, sd for standard deviation. *estimated using ROS to take into account censored data (Helsel, 2012).

MORVAN	Cd* $mg \cdot kg^{-1}$	Cu $mg \cdot kg^{-1}$	Pb $mg \cdot kg^{-1}$	Zn $mg \cdot kg^{-1}$	CEVENNES	Cd* $mg \cdot kg^{-1}$	Cu $mg \cdot kg^{-1}$	Pb $mg \cdot kg^{-1}$	Zn $mg \cdot kg^{-1}$
LOD	*0.5*	*1*	*2*	*2*	*LOD*	*0.5*	*1*	*2*	*2*
**M0**					**C0**				
Min	< 0.5	2	34	35	Min	< 0.5	4	45	37
Q1	***All***	4	44.75	47.5	Q1	***All***	5	57.5	50
Median	***data***	5.5	49.5	61.5	Median	***data***	5	63.5	55
Mean	***below***	6.2	53.25	61.25	Mean	***below***	6.2	63	59.6
Q3	***LOD***	8.25	60.5	71.25	Q3	***LOD***	7	67	63
Max	< 0.5	11	90	90	Max	< 0.5	11	85	107
n.cen	24	0	0	0	n.cen	16	0	0	0
% cen	100	-	-	-	% cen	100	-	-	-
sd	-	-	-	-	sd	-	-	-	-
**M1**					**C1**				
Min	< 0.5	6	41	31	Min	< 0.5	4	47	48
Q1	-	12	47	68.5	Q1	-	8	63	74
Median	0.16	16	129	86	Median	0.15	10	82	94.5
Mean	0.35	29.4	377.2	134.4	Mean	0.85	17	215	224
Q3	-	30	274.2	128.6	Q3	-	12	115	122.5
Max	3.2	212	4520	835	Max	6.8	132	1580	1560
n.cen	73	0	0	0	n.cen	11	0	0	0
% cen	76	-	-	-	% cen	61	-	-	-
sd	0.53	-	-	-	sd	1.8	-	-	-
**M2**					**C2**				
Min	< 0.5	2	64	82	Min	< 0.5	3	31	25
Q1		16	129	210.2	Q1	***All***	3	42	33
Median	1	24.5	1115	338.5	Median	***but one***	4	64	38
Mean	2.4	29.8	1608	641.2	Mean	***below***	21	940	48.7
Q3		43.75	2408	751.8	Q3	***LOD***	5	96	49
Max	54.2	81	8410	13800	Max	0.5	105	4810	142
n.cen	12	0	0	0	n.cen	16	0	0	0
% cen	13	-	-	-	% cen	94	-	-	-
sd	5.85	-	-	-	sd	-	-	-	-

References

Helsel, D.R. *Statistics for censored environmental data using Minitab and R*. Wiley series in statistics in practice (John Wiley & Sons, USA, 2012).

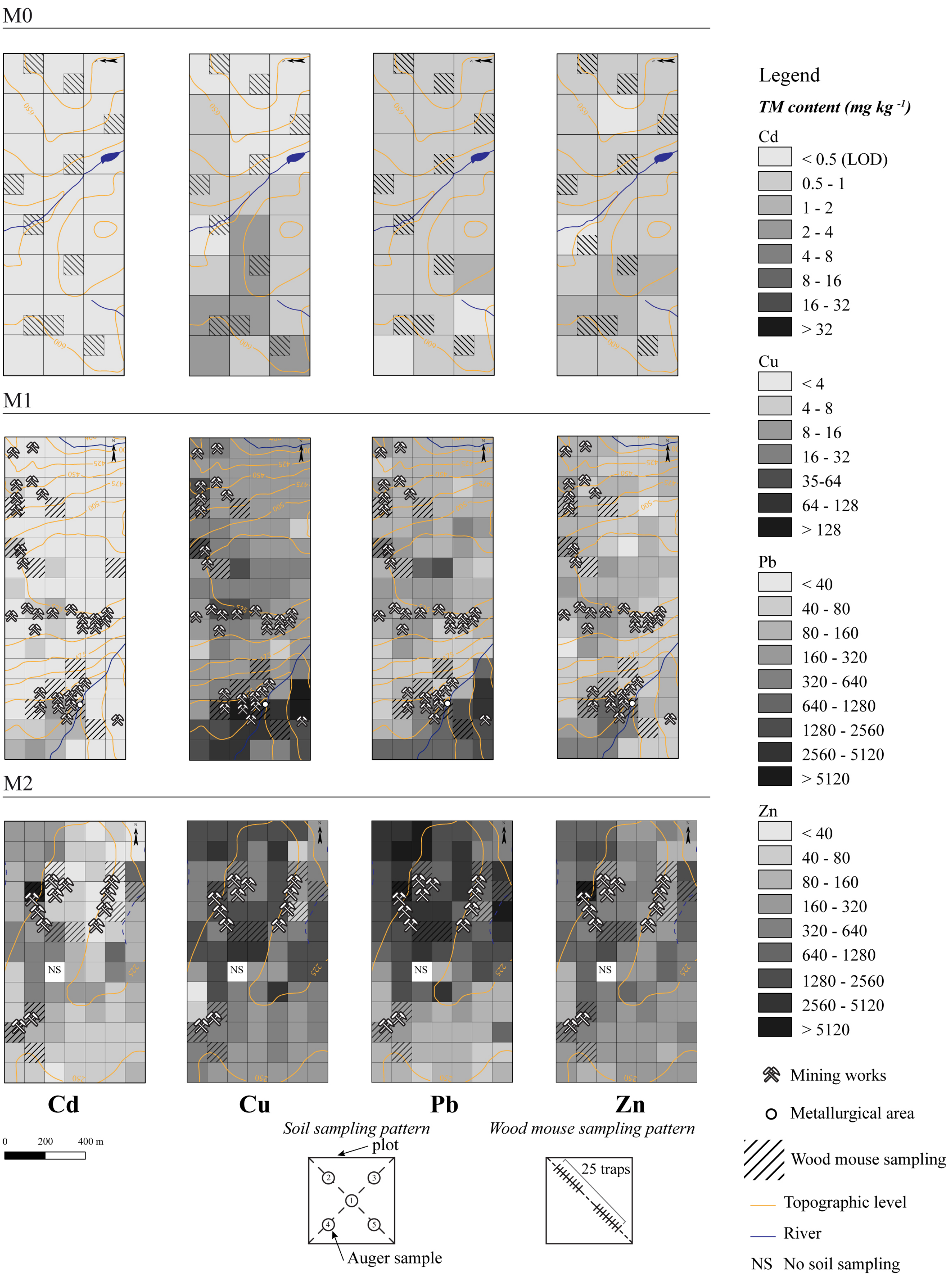

Supplementary Figure S 1 – **Spatial distribution of TMs in Morvan topsoils.** The maps were created using QGIS software (QGIS Essen 2.14.6, http://www.qgis.org). Adapted by E. Camizuli.

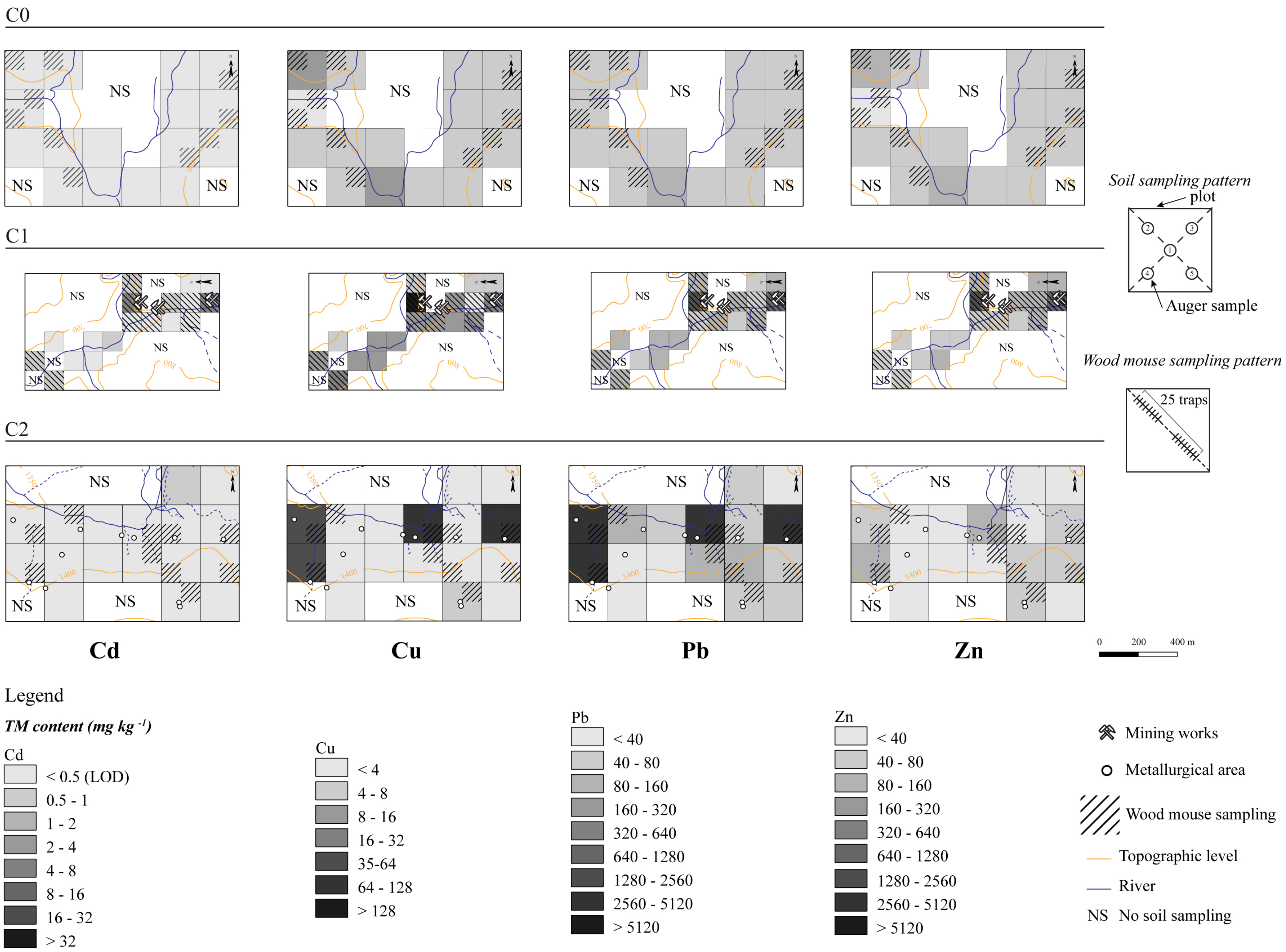

Supplementary Figure S 2 – Spatial distribution of TMs in Cévennes topsoils. The maps were created using QGIS software (QGIS Essen 2.14.6, http://www.qgis.org). Adapted by E. Camizuli.

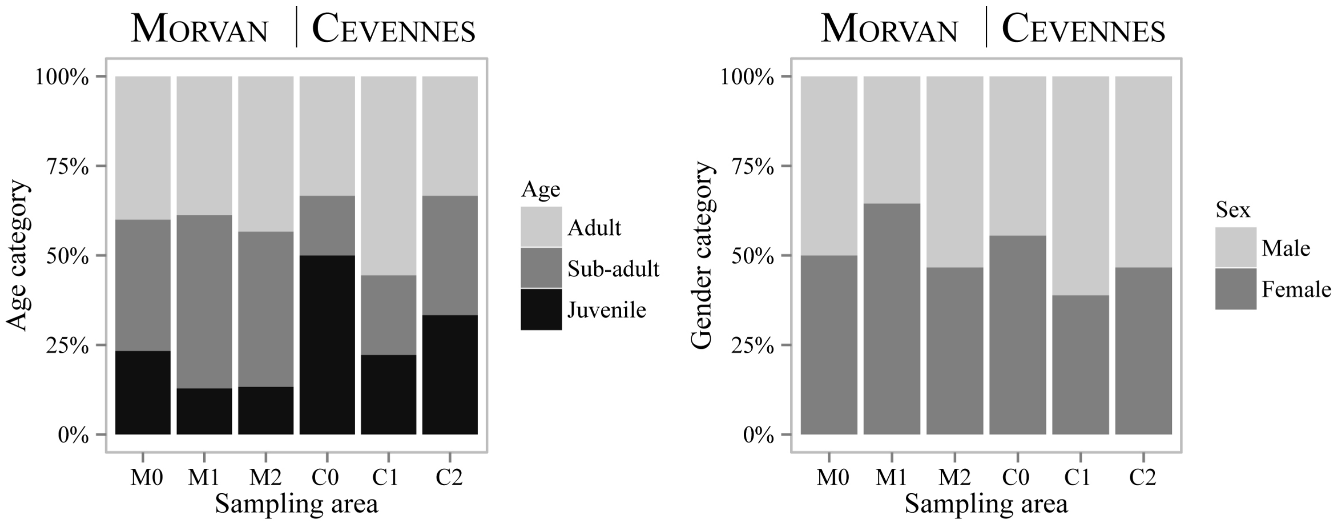

Supplementary Figure S 3 – Age ratio and sex ratio of wood mouse populations.

Supplementary Table S 2 – **Characteristics of wood mice sampled on the six study sites.** For TM concentrations and condition indices, range is indicated with the median between parentheses. Body condition and somatic indices were calculated according to the standard major axis (SMA) regression, SMI stands for Scaled Mass Index, SLI for Scaled Liver Index, and SKI for Scaled Kidney Index. [a]calculated on the entire dataset excluding pregnant females ($n = 154$). [b]calculated on the entire dataset excluding outliers ($n = 155$).

**MORVAN**	M0	M1	M2
Degree of contamination	-	+	++
n	30	31	30
Age			
Juvenile	7	4	4
Sub-Adult	11	15	13
Adult	12	12	13
Sex			
Female	15	20	14
Male	15	11	16
Weight (g)	11.31-35.70 (19)	14.43-26.79 (19.41)	13.36-27.32 (20.61)
Length (mm)	73.55-105.66 (86.25)	77.89-97.31 (86.01)	78.44-101.05 (91.23)
Liver (w. w. g)	0.53-1.59 (1.10)	0.65-1.52 (0.94)	0.54-1.49 (0.99)
Kidneys (w. w. g)	0.17-0.37 (0.24)	0.17-0.33 (0.23)	0.15-0.37 (0.26)
TMs in kidneys ($\mu g \cdot g^{-1}$)			
Cd	0.34-4.64 (1.36)	0.5499-8.93 (2.79)	0.173-26.8 (2.94)
Cu	10.37-21.40 (17.73)	11.85-21.28 (16.54)	12.36-21.61 (17.26)
Pb	0.11-1.71 (0.31)	0.05-2.53 (0.53)	0.1453-5.021 (1.2164)
Zn	44.49-174.5 (83.75)	61.3-133.79 (75.72)	64.89-168.04 (82.98)
Condition indices (g)			
SMI[a]	14.74-31.47 (19.80)	15.81-32.30 (19.67)	13.66-28.50 (17.72)
SLI[b]	0.32-2.05 (1.10)	0.67-2.30 (0.95)	0.60-1.37 (0.88)
SKI[b]	0.13-0.38 (0.26)	0.17-0.42 (0.24)	0.17-0.51 (0.22)
**CEVENNES**	C0	C1	C2
Degree of contamination	-	+	++
n	18	18	30
Age			
Juvenile	9	4	10
Sub-Adult	3	4	10
Adult	6	10	10
Sex			
Female	10	7	14
Male	8	11	16
Weight (g)	9.11-26.8 (16.04)	8.15-35 (24)	11.6-32 (18)
Length (mm)	68-98 (85.3)	68-102 (91)	72-99 (87)
Liver (w. w. g)	0.50-1.80 (0.92)	0.35-2.21 (1.13)	0.66-2.15 (1.18)
Kidney (w. w. g)	0.15-0.47 (0.25)	0.12-0.52 (0.30)	0.18-0.50 (0.26)
TMs in kidneys ($\mu g \cdot g^{-1}$)			
Cd	0.05-1.8 (0.3)	0.1-38 (1.97)	0.07-4 (0.3)
Cu	15.22-21.18 (18)	14.3-20.6 (16.9)	14.7-22 (18.5)
Pb	0.13-0.9 (0.4)	0.07-3.6 (0.25)	0.24-19 (1.4)
Zn	63.48-124.63 (74.6)	55.7-157 (75)	60-160 (81)
Condition indices (g)			
SMI[a]	13.99-24.20 (18.03)	15.10-28.70 (20.67)	13.78-25.57 (19.85)
SLI[b]	0.63-1.78 (1.11)	0.72-1.64 (1.04)	0.74-1.99 (1.29)
SKI[b]	0.19-0.47 (0.30)	0.19-0.42 (0.25)	0.20-0.40 (0.29)

Supplementary Table S 3 – **Parameters of ANOVA tests for trace metal concentrations in wood mouse kidneys.** Summary for the models relating TM concentrations in kidneys to biological and environmental parameters. $0 < *** < 0.001 < ** < 0.01 < * < 0.05 < . < 0.1$.

Models	*coef ANOVA (95% confidence interval)*	*p-value ANOVA*	R^2	$adj-R^2$
$\log_{10}(Cd_{kidneys}) \sim site + mass$			**0.23**	**0.20**
$M2$	$-0.025(-0.260/0.210)$	0.83		
$C1$	$-0.384(-0.665/-0.102)$	$0.008**$		
$mass$	$0.051(0.028/0.075)$	$4.24 \cdot 10^{-05}***$		
$\log_{10}(Cu_{kidneys}) \sim site + sex + mass$			**0.23**	**0.19**
$M1$	$-0.026(-0.051/-0.002)$	$0.04*$		
$M2$	$-0.011(-0.035/0.014)$	0.40		
$C0$	$0.008(-0.021/0.037)$	0.6		
$C1$	$-0.007(-0.036/0.022)$	0.6		
$C2$	$0.014(-0.011/0.039)$	0.3		
$male$	$0.018(0.002/0.034)$	$0.02*$		
$mass$	$-0.004(-0.005/-0.002)$	$9.51 \cdot 10^{-06}***$		
$\log_{10}(Pb_{kidneys}) \sim site + \log_{10}(Pb_{soil})$			**0.35**	**0.33**
$M1$	$-0.055(-0.277/0.168)$	0.6		
$M2$	$0.126(-0.170/0.422)$	0.4		
$C0$	$-0.009(-0.230/0.212)$	0.9		
$C1$	$-0.184(-0.423/0.055)$	0.1		
$C2$	$0.492(0.288/0.696)$	$4.43 \cdot 10^{-06}***$		
$\log_{10}(Pb_{soil})$	$0.234(0.083/0.386)$	$2.67 \cdot 10^{-03}**$		
$\log_{10}(Zn_{kidneys}) \sim site + mass$			**0.14**	**0.10**
$M1$	$-0.069(-0.125/-0.013)$	$0.015*$		
$M2$	$0.007(-0.049/0.063)$	0.8		
$C0$	$-0.078(-0.144/-0.012)$	$0.02*$		
$C1$	$-0.035(-0.100/0.030)$	0.29		
$C2$	$-0.036(-0.092/0.020)$	0.21		
$mass$	$-0.007(-0.010/-0.003)$	$2.69 \cdot 10^{-04}***$		

Supplementary Table S 4 – Parameters of ANOVA tests for body condition and somatic indices. Summary for the models relating body condition and somatic indices to biological and environmental parameters and trace metal concentrations in wood mouse kidneys. $0 < *** < 0.001 < ** < 0.01 < * < 0.05 < . < 0.1$.

Models	*coef ANOVA (95% confidence interval)*	*p-value ANOVA*	R^2	$adj-R^2$
$SMI \sim site + \log_{10} Cd + \log_{10} Cu + \log_{10} Pb + \log_{10} Zn + sex + \log_{10} Cu \times sex + \log_{10} Zn \times sex$			**0.24**	**0.18**
$M1$	$-1.168(-2.850/0.515)$	0.17		
$M2$	$-1.579(-3.396/0.237)$	0.09.		
$C0$	$-2.006(-4.024/0.013)$	0.05.		
$C1$	$0.563(-1.350/2.476)$	0.56		
$C2$	$1.051(-1.011/3.112)$	0.32		
$\log_{10}(Cd_{kidneys})$	$1.285(0.098/2.473)$	$0.03*$		
$\log_{10}(Pb_{kidneys})$	$-1.686(-3.095/-0.277)$	$0.02*$		
$\log_{10}(Cu_{kidneys}) \times sex$	$-37.109(-59.455/-14.762)$	$0.001**$		
$\log_{10}(Zn_{kidneys}) \times sex$	$17.752(7.380/28.123)$	$9.26 \cdot 10^{-04}***$		
$SLI \sim site + \log_{10} Cu + \log_{10} Zn + sex + \log_{10} Cu \times sex + \log_{10} Zn \times sex$			**0.30**	**0.25**
$M1$	$-0.201(-0.342/-0.059)$	$0.006**$		
$M2$	$-0.248(-0.391/-0.104)$	$8.22 \cdot 10^{04}***$		
$C0$	$-0.065(-0.230/0.099)$	0.43		
$C1$	$-0.080(-0.244/0.084)$	0.34		
$C2$	$0.149(0.004/0.294)$	$0.04*$		
$\log_{10}(Cu_{kidneys}) \times sex$	$-3.039(-4.934/-1.144)$	$0.002**$		
$\log_{10}(Zn_{kidneys}) \times sex$	$1.110(0.205/2.015)$	$0.02*$		
$SKI \sim site + \log_{10} Cu + \log_{10} Zn + sex + \log_{10} Cu \times sex + \log_{10} Zn \times sex$			**0.25**	**0.20**
$M1$	$-0.029(-0.058/0.001)$	0.06.		
$M2$	$-0.025(-0.055/0.005)$	0.10.		
$C0$	$0.029(-0.006/0.063)$	0.11		
$C1$	$0.020(-0.015/0.054)$	0.27		
$C2$	$0.036(0.0060.067)$	$0.02*$		
$\log_{10}(Cu_{kidneys}) \times sex$	$-0.673(-1.075/-0.271)$	$0.001**$		
$\log_{10}(Zn_{kidneys}) \times sex$	$0.310(0.121/0.499)$	$0.001**$		

Supplementary Table S 5 – Details of preliminary tests performed before treating fluctuating asymmetry for the six wood mouse traits studied. LM1 stands for length of the first lower molar, LM2 for length of the second lower molar, LM3 for length of the third lower molar, WM1 for width of the first lower molar, WM2 for width of the second lower molar, and WM3 for width of the third lower molar; R for right side and L for left side; K-S for Kolmogorov Smirnov, Skew for skewness, Kurt for kurtosis; $FA4 = var(R-L)$ and $FA1 = mean|R-L|$.[a]Values x 10^5. [b]Values x 10^3.

			Character size		Asymmetry																
			$(R+L)/2$		$\|R-L\| vs.(R+L)/2$		$(R-L)$													$\|R-L\|$	
**Trait**	**Sample**	N	**Mean**	$\pm SE$	**Slope**	p	**Mean**	$\pm SE$	**t_test**	p	**Swilk W**	p	**K-S Dist**	p	**Skew**	p	**Kurt**	p	$FA4^a$	$FA1^b$	$\pm SE$
LM1	M0	39	1.762	0.014	0.010	0.67	0.004	0.003	1.45	0.16	0.98	0.67	0.11	0.28	0.25	0.64	2.52	0.68	33.4	14.3	0.002
LM1	M1	36	1.742	0.012	-0.012	0.60	-0.001	0.003	-0.23	0.82	0.94	**0.04**	0.13	0.16	-0.63	0.27	2.52	0.72	25.1	12.4	0.002
LM1	M2	26	1.720	0.014	-0.103	**0.0009**	0.002	0.004	0.43	0.67	0.98	0.93	0.08	0.96	0.04	0.95	2.32	0.56	41.2	16.2	0.002
LM1	C0	11	1.737	0.023	0.060	0.20	-0.001	0.007	-0.20	0.85	0.91	0.24	0.18	0.40	0.41	0.62	1.81	0.32	54.7	19.8	0.003
LM1	C1	13	1.767	0.022	-0.052	0.15	0.001	0.004	0.31	0.76	0.97	0.84	0.13	0.78	0.47	0.56	3.48	0.22	21.2	10.4	0.003
LM1	C2	25	1.726	0.015	-0.050	**0.04**	0.002	0.004	0.45	0.65	0.93	0.07	0.20	**0.02**	-0.35	0.58	1.83	0.06	42.0	18.2	0.002
LM2	M0	39	1.217	0.009	0.028	0.33	0.002	0.003	0.58	0.56	0.98	0.80	0.10	0.42	0.27	0.61	2.50	0.66	33.0	14.9	0.002
LM2	M1	36	1.193	0.008	0.002	0.96	-0.004	0.003	-1.26	0.22	0.97	0.34	0.10	0.51	-0.39	0.48	2.57	0.79	41.9	16.9	0.002
LM2	M2	26	1.179	0.013	-0.005	0.88	-0.0003	0.004	-0.08	0.93	0.96	0.43	0.12	0.40	-0.55	0.38	2.80	0.79	40.7	16.6	0.002
LM2	C0	11	1.203	0.015	-0.074	0.39	0.007	0.006	1.14	0.28	0.91	0.27	0.21	0.22	-0.98	0.25	3.72	0.14	43.3	17.2	0.004
LM2	C1	13	1.219	0.014	0.008	0.91	0.006	0.005	1.33	0.21	0.93	0.38	0.17	0.41	0.37	0.64	1.91	0.36	30.6	14.2	0.003
LM2	C2	25	1.202	0.008	0.059	0.28	0.011	0.003	3.51	**0.002**	0.96	0.41	0.17	0.08	-0.22	0.73	2.06	0.24	23.6	15.6	0.002
LM3	M0	39	1.015	0.007	-0.056	0.21	0.00001	0.003	0.00	1.00	0.98	0.85	0.07	0.92	0.13	0.80	3.18	0.47	40.7	15.6	0.002
LM3	M1	36	0.972	0.009	0.060	0.27	-0.001	0.005	-0.20	0.84	0.98	0.71	0.08	0.74	-0.30	0.59	3.39	0.33	82.0	22.6	0.003
LM3	M2	26	0.981	0.008	-0.106	**0.04**	-0.004	0.003	-1.07	0.30	0.96	0.44	0.12	0.37	-0.38	0.54	2.52	0.85	31.3	13.8	0.002
LM3	C0	11	1.005	0.015	-0.015	0.86	-0.013	0.008	-1.65	0.13	0.88	0.10	0.22	0.14	0.38	0.64	1.60	0.13	65.7	25.0	0.004
LM3	C1	13	1.003	0.016	-0.125	**0.03**	-0.006	0.008	-0.76	0.46	0.93	0.35	0.18	0.28	0.23	0.77	1.71	0.16	76.2	24.5	0.003
LM3	C2	25	1.022	0.011	0.079	0.24	0.013	0.005	2.69	**0.01**	0.94	0.13	0.12	0.53	-0.59	0.36	3.54	0.25	59.4	21.1	0.004
WM1	M0	39	1.067	0.006	0.055	0.31	-0.012	0.003	-3.83	**0.0005**	0.98	0.78	0.11	0.24	0.25	0.64	3.21	0.45	35.2	18.2	0.002
WM1	M1	36	1.061	0.007	0.034	0.50	-0.008	0.003	-2.96	**0.01**	0.96	0.25	0.12	0.25	-0.61	0.28	3.81	0.16	25.8	13.4	0.002
WM1	M2	26	1.066	0.011	0.049	0.22	-0.010	0.003	-3.32	**0.003**	0.99	0.97	0.08	0.96	-0.19	0.76	2.62	0.98	23.5	14.3	0.002
WM1	C0	11	1.061	0.018	-0.003	0.96	-0.005	0.006	-0.75	0.47	0.88	0.10	0.25	0.05	1.01	0.24	2.94	0.45	41.1	17.5	0.003
WM1	C1	13	1.088	0.012	-0.043	0.68	-0.014	0.004	-3.24	**0.01**	0.88	0.06	0.22	0.10	-1.08	0.20	3.28	0.30	25.5	15.3	0.004
WM1	C2	25	1.041	0.010	-0.083	0.16	-0.012	0.004	-3.07	**0.01**	0.97	0.76	0.12	0.46	0.06	0.93	2.22	0.43	38.7	18.4	0.003
WM2	M0	39	1.070	0.007	0.006	0.83	0.001	0.002	0.42	0.68	0.95	0.08	0.11	0.26	-0.81	0.15	4.61	**0.04**	13.0	8.2	0.001
WM2	M1	36	1.066	0.007	-0.004	0.87	0.004	0.002	2.14	**0.04**	0.96	0.29	0.19	**0.003**	-0.33	0.54	2.57	0.80	11.1	9.6	0.001
WM2	M2	26	1.060	0.009	0.053	0.31	0.007	0.003	2.23	**0.03**	0.94	0.16	0.17	0.05	0.40	0.52	4.17	0.09	23.3	11.9	0.002
WM2	C0	11	1.066	0.013	-0.065	0.35	0.003	0.004	0.84	0.42	0.97	0.84	0.18	0.45	0.53	0.52	2.60	0.73	16.7	9.6	0.003
WM2	C1	13	1.094	0.013	-0.003	0.95	-0.005	0.003	-1.63	0.13	0.97	0.90	0.15	0.56	-0.31	0.70	2.15	0.66	10.5	8.8	0.002
WM2	C2	25	1.051	0.009	0.004	0.95	0.007	0.004	1.83	0.08	0.96	0.52	0.12	0.50	-0.16	0.79	3.42	0.30	36.4	15.2	0.003
WM3	M0	39	0.888	0.007	0.051	**0.03**	-0.0002	0.002	-0.11	0.91	0.97	0.47	0.09	0.61	-0.01	0.98	2.22	0.24	14.3	10.0	0.001
WM3	M1	36	0.874	0.006	0.043	0.33	0.006	0.002	2.34	**0.03**	0.98	0.61	0.09	0.73	-0.05	0.92	2.94	0.70	20.2	11.6	0.002
WM3	M2	26	0.881	0.008	0.017	0.66	0.003	0.003	1.23	0.23	0.95	0.24	0.12	0.46	0.56	0.38	2.58	0.93	16.6	10.4	0.002
WM3	C0	11	0.875	0.012	0.124	0.09	0.0002	0.004	0.04	0.97	0.95	0.66	0.15	0.66	-0.25	0.76	2.35	1.00	21.0	10.6	0.003
WM3	C1	13	0.909	0.015	-0.018	0.58	-0.002	0.003	-0.71	0.49	0.95	0.63	0.14	0.71	-0.23	0.77	1.94	0.39	13.0	9.6	0.002
WM3	C2	25	0.893	0.009	0.012	0.77	-0.001	0.002	-0.40	0.69	0.95	0.28	0.13	0.32	-0.24	0.70	3.19	0.43	15.3	8.9	0.002

Supplementary Table S 6 – **Summary results for the linear regression of —right-left— versus internal concentration in wood mouse kidneys, for each trait studied.** LM1 stands for length of the first lower molar, LM2 for length of the second lower molar, LM3 for length of the third lower molar, WM1 for width of the first lower molar, WM2 for width of the second lower molar, and WM3 for width of the third lower molar.

Trait	*n*	log_{10}(Cd) Slope	*p*	log_{10}(Cu) Slope	*p*	log_{10}(Pb) Slope	*p*	log_{10}(Zn) Slope	*p*
LM1	131	-0.002	0.22	0.017	0.33	0.003	0.12	-0.001	0.87
LM2	131	0.001	0.72	-0.020	0.24	0.0004	0.86	-0.003	0.71
LM3	131	-0.002	0.28	0.022	0.36	-0.002	0.58	0.012	0.27
WM1	131	0.001	0.72	0.003	0.86	-0.0001	0.97	-0.004	0.69
WM2	131	-0.002	0.14	0.009	0.54	0.002	0.22	-0.005	0.47
WM3	131	-0.001	0.46	-0.004	0.77	-0.001	0.64	-0.005	0.35

Supplementary Table S 7 – **Results of the two-way mixed model ANOVA (side fixed x individual random) with repeated measurement on each side.** LM1 stands for length of the first lower molar, LM2 for length of the second lower molar, LM3 for length of the third lower molar, WM1 for width of the first lower molar, WM2 for width of the second lower molar, and WM3 for width of the third lower molar.[a]Values x 10^5.

		M0						M1						M2					
		MS	*df*	*F*	*p*	$FA10^a$	$df(FA10)$	*MS*	*df*	*F*	*p*	$FA10^a$	$df(FA10)$	*MS*	*df*	*F*	*p*	$FA10^a$	$df(FA10)$
LM1	Side	6.98E−04	1	2.09	0.16			1.34E−05	1	0.05	0.82			7.79E−05	1	0.19	0.82		
	Individual	0.03	38	85.16	2.46E−27			0.02	35	81.93	5.04E−25			0.02	25	49.37	1.13E−15		
	Interaction	3.34E−04	38	8.48	1.43E−15	14.7	29.4	2.52E−04	35	6.37	1.91E−11	10.6	24.6	4.12E−04	25	5.99	2.82E−08	17.2	17.1
	Meas. Error	3.94E−05	78					3.95E−05	72					6.88E−05	52				
LM2	Side	1.12E−04	1	0.34	0.56			6.67E−04	1	1.59	0.22			2.80E−06	1	0.01	0.82		
	Individual	0.01	38	42.65	8.25E−22			0.01	35	21.52	2.42E−15			0.02	25	46.31	2.44E−15		
	Interaction	3.30E−04	38	5.38	1.77E−10	13.4	24.8	4.20E−04	35	6.42	1.56E−11	17.7	24.7	4.08E−04	25	9.29	1.10E−11	18.2	19.8
	Meas. Error	6.13E−05	78					6.53E−05	72					4.39E−05	52				
LM3	Side	0.00E+00	1	0.00	1.00			3.21E−05	1	0.04	0.84			3.58E−04	1	1.14	0.82		
	Individual	0.01	38	20.54	3.65E−16			0.01	35	13.98	2.10E−12			0.01	25	23.94	5.95E−12		
	Interaction	4.07E−04	38	4.05	8.59E−08	15.3	20.9	8.20E−04	35	8.57	1.25E−14	36.2	27.1	3.13E−04	25	3.75	2.88E−05	11.5	13.0
	Meas. Error	1.01E−04	78					9.57E−05	72					8.35E−05	52				
WM1	Side	0.01	1	14.66	4.67E−04			2.26E−03	1	8.79	5.43E−03			2.60E−03	1	11.05	2.74E−03		
	Individual	0.01	38	15.02	7.65E−14			0.01	35	24.95	2.22E−16			0.01	25	56.83	2.07E−16		
	Interaction	3.52E−04	38	4.00	1.11E−07	13.2	20.8	2.58E−04	35	4.53	3.05E−08	10.0	20.8	2.35E−04	25	4.52	2.21E−06	9.2	14.8
	Meas. Error	8.79E−05	78					5.69E−05	72					5.20E−05	52				
WM2	Side	2.31E−05	1	0.18	0.68			5.10E−04	1	4.59	3.92E−02			1.16E−03	1	4.97	3.50E−02		
	Individual	0.01	38	62.28	8.04E−25			0.01	35	59.70	1.11E−22			0.01	25	33.22	1.28E−13		
	Interaction	1.30E−04	38	5.36	1.95E−10	5.3	24.7	1.11E−04	35	4.07	2.38E−07	4.2	19.3	2.33E−04	25	6.16	1.80E−08	9.8	17.3
	Meas. Error	2.42E−05	78					2.73E−05	72					3.78E−05	52				
WM3	Side	1.90E−06	1	0.01	0.91			1.11E−03	1	5.48	2.51E−02			2.49E−04	1	1.50	0.82		
	Individual	0.01	38	52.36	1.95E−23			0.01	35	27.92	3.58E−17			0.01	25	44.31	4.15E−15		
	Interaction	1.43E−04	38	7.23	1.10E−13	6.2	28.0	2.02E−04	35	6.61	7.85E−12	8.6	24.9	1.66E−04	25	6.08	2.26E−08	6.9	17.2
	Meas. Error	1.98E−05	78					3.05E−05	72					2.73E−05	52				

		C0						C1						C2					
		MS	*df*	*F*	*p*	$FA10^a$	$df(FA10)$	*MS*	*df*	*F*	*p*	$FA10^a$	$df(FA10)$	*MS*	*df*	*F*	*p*	*FA10a*	$df(FA10)$
LM1	Side	2.18E−05	1	0.04	0.85			2.09E−05	1	0.10	0.76			8.65E−05	1	0.21	0.65		
	Individual	0.02	10	42.45	7.53E−07			0.03	12	119.74	1.44E−10			0.02	24	52.02	2.26E−15		
	Interaction	5.47E−04	10	14.20	2.05E−07	25.4	8.6	2.12E−04	12	2.23	4.19E−02	5.9	3.4	4.20E−04	24	10.66	1.99E−12	19.0	19.6
	Meas. Error	3.85E−05	22					9.49E−05	26					3.94E−05	50				
LM2	Side	5.60E−04	1	1.29	0.28			5.43E−04	1	1.78	0.21			2.91E−03	1	12.33	1.79E−03		
	Individual	0.01	10	22.24	1.61E−05			0.01	12	35.74	1.67E−07			0.01	24	25.27	8.44E−12		
	Interaction	4.33E−04	10	6.76	9.69E−05	18.4	7.2	3.06E−04	12	4.59	5.61E−04	12.0	7.2	2.36E−04	24	3.25	2.18E−04	8.2	11.0
	Meas. Error	6.40E−05	22					6.66E−05	26					7.25E−05	50				
LM3	Side	1.79E−03	1	2.73	0.13			4.44E−04	1	0.58	0.46			4.29E−03	1	7.22	1.29E−02		
	Individual	0.01	10	15.79	7.71E−05			0.01	12	18.04	7.70E−06			0.01	24	19.60	1.40E−10		
	Interaction	6.57E−04	10	14.51	1.68E−07	30.6	8.7	7.62E−04	12	13.72	2.47E−08	35.3	10.3	5.94E−04	24	5.19	4.64E−07	24.0	15.4
	Meas. Error	4.53E−05	22					5.55E−05	26					1.14E−04	50				
WM1	Side	2.32E−04	1	0.56	0.47			2.68E−03	1	10.49	7.10E−03			3.64E−03	1	9.40	5.30E−03		
	Individual	0.01	10	36.09	1.64E−06			0.01	12	31.03	3.74E−07			0.01	24	23.41	1.97E−11		
	Interaction	4.11E−04	10	7.79	3.30E−05	17.9	7.5	2.55E−04	12	5.10	2.51E−04	10.3	7.6	3.87E−04	24	8.10	3.23E−10	17.0	18.3
	Meas. Error	5.28E−05	22					5.00E−05	26					4.78E−05	50				
WM2	Side	1.18E−04	1	0.70	0.42			2.77E−04	1	2.64	0.13			1.23E−03	1	3.36	0.08		
	Individual	0.01	10	43.00	7.08E−07			0.01	12	85.27	1.07E−09			0.01	24	20.85	7.12E−11		
	Interaction	1.67E−04	10	7.79	3.28E−05	7.3	7.5	1.05E−04	12	3.22	6.10E−03	3.6	5.5	3.64E−04	24	16.69	2.36E−16	17.1	21.2
	Meas. Error	2.15E−05	22					3.26E−05	26					2.18E−05	50				
WM3	Side	4.00E−07	1	0.00	0.97			6.47E−05	1	0.50	0.49			2.50E−05	1	0.16	0.69		
	Individual	0.01	10	29.42	4.33E−06			0.01	12	95.29	5.54E−10			0.01	24	52.23	2.15E−15		
	Interaction	2.10E−04	10	6.95	7.85E−05	9.0	7.3	1.30E−04	12	2.85	1.23E−02	4.2	4.8	1.53E−04	24	5.77	9.36E−08	6.3	16.2
	Meas. Error	3.02E−05	22					4.55E−05	26					2.65E−05	50				

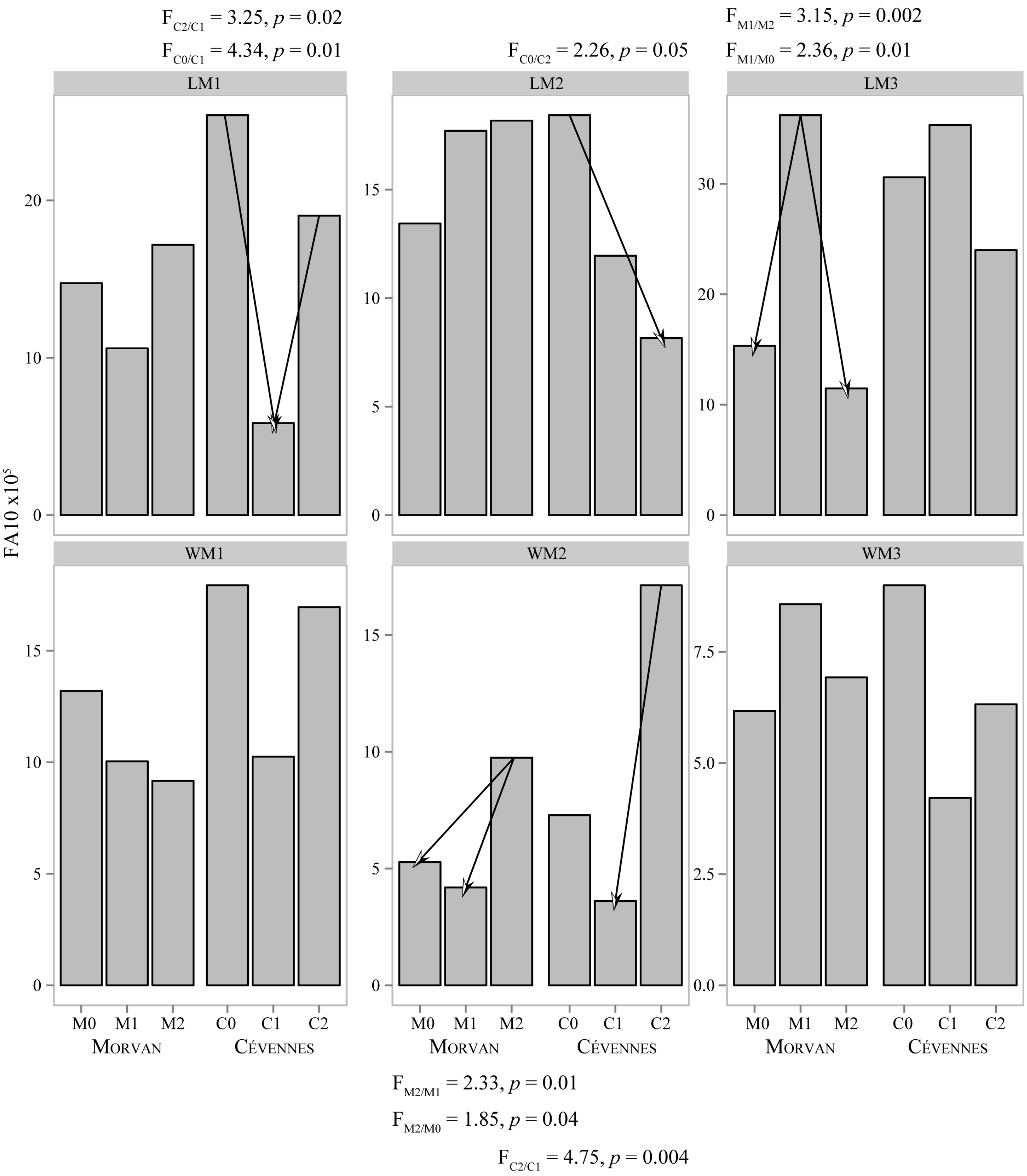

Supplementary Figure S 4 – FA10 values for each study site. LM1 stands for length of the first lower molar, LM2 for length of the second lower molar, LM3 for length of the third lower molar, WM1 for width of the first lower molar, WM2 for width of the second lower molar, and WM3 for width of the third lower molar.

Supplementary Table S 8 – **Quality control of soil analysis for stream sediments (JSD1, JSD2) and marine and estuarine sediments (BCSS1, PACS1).**

	Cd	Cu	Pb	Zn
LOD (mg $\cdot kg^{-1}$)	0.5	1	2	2
JSD1 (mg $\cdot kg^{-1}$)				
a	< 0.5	22	6	93
b	< 0.5	22	24	97
certified		*22.2*	*14*	*99*
JSD2 (mg $\cdot kg^{-1}$)				
a	3.3	1070	133	1860
b	3.6	1080	150	2050
certified	-	*1114*	*151*	*2070*
BCSS1 (mg $\cdot kg^{-1}$)				
a	< 0.5	15	18	110
b	0.9	32	50	222
certified	*0.25*	*18.5*	*22.7*	*119*
PACS1 (mg $\cdot kg^{-1}$)				
a	2.5	397	358	767
b	5.4	852	772	1690
certified	*2.38*	*452*	*404*	*824*

Supplementary Table S 9 – Topsoil results.

ID	Cd	Cu	Pb	Zn	ID	Cd	Cu	Pb	Zn	ID	Cd	Cu	Pb	Zn	ID	Cd	Cu	Pb	Zn	ID	Cd	Cu	Pb	Zn
M0-1	<	11	34	71	M1-29	<	14	439	82	M1-84 #1	<	212	3710	127	M2-43	2.2	26	1520	513	C0-01 #1	<	11	53	81
M0-3 #1	<	6	44	61	M1-30	<	7	165	48	M1-84 #2	<	216	3640	130	M2-44	4.4	33	2890	984	C0-01 #2	<	8	51	79
M0-3 #2	<	7	54	64	M1-31	0.6	41	501	255	M1-84 #3	<	209	3790	125	M2-45	0.9	52	4080	811	C0-03	<	6	45	76
M0-5	<	10	46	69	M1-32	0.5	30	289	134	M1-85	0.6	44	155	261	M2-46	<	28	1490	280	C0-09	<	8	73	55
M0-13	<	11	50	85	M1-33	<	18	106	58	M1-86	0.7	36	158	239	M2-47	0.8	17	700	225	C0-11	<	5	63	50
M0-15	<	10	77	90	M1-34	<	13	113	33	M1-87	2	115	449	671	M2-48	1.3	46	3190	771	C0-26	<	4	58	37
M0-17	<	5	35	59	M1-35	<	12	182	97	M1-88	1.3	65	869	341	M2-50	5	46	1240	793	C0-33	<	7	67	59
M0-25	<	5	48	66	M1-36	<	15	95	72	M1-89	<	62	2170	67	M2-51	5	45	2140	1040	C0-35	<	5	69	49
M0-27	<	11	63	84	M1-37	<	15	144	98	M1-90 #1	<	42	1320	76	M2-52	6.4	65	3450	1470	C0-49	<	5	66	44
M0-29	<	7	90	81	M1-38	<	12	97	62	M1-90 #2	<	40	1100	66	M2-53	1.1	78	2320	570	C0-51	<	5	64	61
M0-37	<	3	42	35	M1-39	0.7	41	884	315	M1-91	1.3	59	645	373	M2-54 #1	0.6	18	726	221	C0-53	<	6	57	57
M0-39	<	9	80	80	M1-40 #1	<	31	1400	137	M1-92	2.4	90	898	587	M2-54 #2	<	16	477	201	C0-57	<	5	61	51
M0-41 #1	<	8	65	54	M1-40 #2	<	36	1280	143	M1-93	1.7	77	746	459	M2-55	10.3	44	1630	1260	C0-72 #1	<	5	64	47
M0-41 #2	<	8	68	59	M1-40 #3	<	30	1280	139	M1-94	<	18	667	46	M2-56	1.3	37	2560	471	C0-72 #2	<	5	64	47
M0-41 #3	<	8	61	49	M1-40 #4	0.5	33	1280	141	M1-95	<	29	985	51	M2-58	1	11	205	120	C0-72 #3	<	5	64	46
M0-49	<	6	41	62	M1-41	<	25	116	87	M1-96	<	50	1380	60	M2-59	5.5	54	2340	1060	C0-75	<	5	55	55
M0-51	<	5	52	41	M1-42	<	30	79	109	M2-2	2.5	59	3290	769	M2-61	1.1	16	83	183	C0-77	<	10	85	107
M0-53	<	8	60	72	M1-43	0.7	24	275	165	M2-3	2.5	48	4240	806	M2-62	0.8	16	482	233	C0-79	<	7	67	69
M0-61	<	6	62	57	M1-44	0.5	17	308	117	M2-4 #1	3.8	62	5530	1190	M2-63	4.2	44	2080	965	C0-81 #1	<	5	62	55
M0-63	<	4	45	42	M1-45	<	22	273	97	M2-4 #2	3.7	61	> 5000	1190	M2-64	2.3	34	950	335	C0-81 #2	<	5	65	58
M0-65	<	2	41	51	M1-46	<	18	319	88	M2-4 #3	3.9	64	> 5000	1200	M2-66	0.7	2	90	82	C1-03	0.6	14	71	101
M0-73	<	5	49	70	M1-47	<	18	123	90	M2-5 #1	0.7	52	1420	290	M2-67	5.2	40	1600	1040	C1-12	0.7	19	77	128
M0-75 #1	<	3	47	40	M1-48	<	15	154	69	M2-5 #2	0.7	53	1430	293	M2-68	2.4	25	823	629	C1-14	<	7	67	100
M0-75 #2	<	3	49	42	M1-49	0.5	15	199	117	M2-5 #3	0.7	51	1400	287	M2-69 #1	3.6	31	2790	912	C1-23 #1	<	12	47	60
M0-77	<	3	53	63	M1-50	0.6	42	110	200	M2-6	<	47	1590	293	M2-69 #2	3.4	32	2710	880	C1-23 #2	<	12	47	60
M0-85	<	4	52	46	M1-51	1.1	39	274	267	M2-7	0.8	56	1170	377	M2-70	2.5	66	449	479	C1-23 #3	<	12	47	59
M0-87	<	3	45	43	M1-52	<	18	179	87	M2-8	0.5	13	391	201	M2-71	0.8	17	132	214	C1-33 #1	<	10	88	95
M0-89	<	4	57	48	M1-53	<	11	98	73	M2-10	2.4	41	4280	774	M2-72 #1	1.3	21	132	254	C1-33 #2	0.5	10	93	95
M1-1 #1	<	28	245	116	M1-54 #1	<	14	130	63	M2-11 #1	3.5	75	8410	1720	M2-72 #2	1.2	21	132	252	C1-34	<	10	62	94
M1-1 #2	<	29	244	117	M1-54 #2	<	16	131	71	M2-11 #2	2.2	53	> 5000	1220	M2-72 #3	1.3	21	133	256	C1-44	0.6	10	62	91
M1-1 #3	<	27	247	115	M1-55	<	10	105	49	M2-11 #3	1.9	55	> 5000	1330	M2-74	0.9	5	98	136	C1-55-2	<	11	56	66
M1-2	<	21	146	86	M1-56	0.5	27	170	239	M2-12	8	50	7380	2290	M2-75	3.2	20	274	700	C1-56	6.8	132	1580	1560
M1-3 #1	<	10	108	82	M1-57	0.7	28	251	230	M2-13	0.9	32	1580	367	M2-76	1.4	21	237	342	C1-57	0.7	8	127	159
M1-3 #2	<	10	101	79	M1-58	<	13	75	82	M2-14	0.8	81	4970	559	M2-77	0.7	15	151	207	C1-65	<	11	76	92
M1-4	<	11	88	72	M1-59 #1	<	10	74	67	M2-15	<	8	629	103	M2-78	1.4	23	222	331	C1-75 #1	<	12	56	71
M1-5	<	15	83	74	M1-59 #2	<	10	77	69	M2-16	<	11	383	125	M2-79	0.7	16	96	238	C1-75 #2	<	15	72	89
M1-6	<	15	124	112	M1-59 #3	<	9	70	65	M2-18	1.2	31	2320	449	M2-80 #1	1.2	20	114	226	C1-76-2	0.6	9	136	109
M1-7	<	10	66	155	M1-60	<	14	129	70	M2-19	3	31	5120	937	M2-80 #2	1.2	20	123	230	C1-85	<	10	106	84
M1-8	<	16	111	106	M1-61	<	8	75	34	M2-20 #1	<	20	1210	265	M2-82	2.4	24	211	629	C1-86 #1	<	4	95	48
M1-9 #1	<	9	109	70	M1-62	0.6	31	196	255	M2-20 #2	<	20	1230	272	M2-83	1.6	20	176	351	C1-86 #2	<	4	99	51
M1-9 #2	<	10	98	81	M1-63	<	16	124	116	M2-20 #3	0.5	20	1190	257	M2-84	1.2	20	112	303	C1-86 #3	<	3	91	46
M1-10	<	9	82	70	M1-64	<	12	65	70	M2-21	0.5	22	1140	300	M2-85	0.6	15	114	181	C1-87	<	8	115	56
M1-11	<	9	102	55	M1-65	<	6	41	58	M2-22 #1	0.7	55	3950	605	M2-86	1	19	112	207	C1-96-2	4.5	20	937	999
M1-12	<	12	97	106	M1-66 #1	<	10	57	52	M2-22 #2	<	52	3690	567	M2-87 #1	1	16	86	216	C1-97	<	5	116	127
M1-13 #1	0.9	60	187	223	M1-66 #2	<	10	57	51	M2-23	<	9	995	141	M2-87 #2	0.9	16	84	208	C2-10	0.5	3	52	25
M1-13 #2	0.9	60	192	223	M1-67	<	14	74	55	M2-24	8.4	29	2680	1530	M2-87 #3	1.1	17	88	225	C2-12	<	3	32	34
M1-13 #3	0.9	61	182	222	M1-68	<	10	79	55	M2-26 #1	1.6	25	1760	444	M2-88	1	22	957	358	C2-28	<	52	3560	59
M1-14	<	12	100	75	M1-69	<	13	80	82	M2-26 #2	1.4	24	1670	411	M2-90	1	19	119	233	C2-30	<	4	96	36
M1-15	<	11	118	83	M1-70	<	17	96	75	M2-27	48.7	49	5340	13800	M2-91	0.7	18	127	221	C2-32	<	4	64	33
M1-16	<	10	98	82	M1-71 #1	<	9	63	66	M2-28	<	25	1700	323	M2-92	0.7	16	104	191	C2-34	<	105	4810	83
M1-17	<	12	64	50	M1-71 #2	<	8	63	62	M2-29	0.7	30	1580	481	M2-93 #1	1	15	117	169	C2-36 #1	<	4	79	38
M1-18	<	11	90	94	M1-72	<	21	676	112	M2-30	<	33	2580	351	M2-93 #2	0.8	16	107	173	C2-36 #2	<	5	86	41
M1-19 #1	0.5	28	122	93	M1-73	<	12	80	55	M2-31	<	12	1090	200	M2-94	0.9	17	94	175	C2-52	<	90	2670	68
M1-19 #2	<	24	122	95	M1-74	0.5	17	150	160	M2-32	3.9	27	2430	918	M2-95	0.8	14	64	155	C2-56 #1	<	3	38	32
M1-20	<	15	116	87	M1-75 #1	<	22	93	104	M2-34	1.6	24	2010	349	M2-96	0.9	15	74	286	C2-56 #2	<	3	37	30
M1-21	<	12	159	78	M1-75 #2	0.6	22	101	118	M2-35	4.8	33	2890	1020	M2-98	1	18	113	209	C2-56 #3	<	3	39	34
M1-22	<	12	147	74	M1-76	<	17	66	70	M2-36	0.8	40	2760	556	M2-99	0.6	14	127	159	C2-60	<	3	82	37
M1-23	<	13	126	31	M1-77	<	10	100	50	M2-37	0.9	43	3660	633	M2-100 #1	<	10	73	102	C2-62	<	4	95	46
M1-24	<	11	129	69	M1-78	<	155	4520	149	M2-38 #1	<	33	1180	180	M2-100 #2	<	11	76	107	C2-64	<	4	55	49
M1-25	<	17	109	97	M1-79	<	13	65	57	M2-38 #2	<	33	1180	179	M2-101 #1	0.8	15	115	218	C2-68	<	61	4180	142
M1-26 #1	<	18	140	86	M1-80	<	40	133	197	M2-38 #3	<	34	1170	181	M2-101 #2	0.7	15	84	213	C2-72	<	3	37	32
M1-26 #2	<	17	138	85	M1-81	3.2	145	455	835	M2-39	<	6	311	111	M2-101 #3	0.9	15	146	223	C2-82	<	3	31	30
M1-26 #3	<	18	142	87	M1-82 #1	1.8	120	1110	499	M2-40	1.5	63	6130	992	M2-102	0.7	15	128	165	C2-88	<	5	59	44
M1-27	<	15	196	73	M1-82 #2	2	117	1130	502	M2-42 #1	4.1	44	2150	699	M2-103	0.8	14	68	161	C2-90	<	4	42	40
M1-28	<	14	99	66	M1-83	<	110	2440	114	M2-42 #2	3.9	41	1880	610	M2-104	1.3	16	121	281					

Supplementary Table S 10 – **Use of body weight to calculate wood mouse age.** Body weight is the most commonly used indicator of age for small mammals (Gonzalez et al., 2008). Body wet weight was used as an index of age even though this technique suffers from the variable influence of diet, stress and metabolic condition (Hunter et al., 1987, 1984 in Milton et al., 2003). Specimens were divided into three age classes: juvenile (J), sub-adult (SA), and adult (A) by combining body weight and sexual activity traits for females (placenta scars). We used arbitrary ranges of body weight for each sex as described by Peig and Green (2010).

**Mass in** *g*	**Juvenile**	**Sub-Adult**	**Adult**
Male	< 17	$17 - 22$	$>= 22$
Female	< 16	$16 - 21$	$>= 21$

References

González, X. I., Aboal, J. R., Fernández, J. A. & Carballeira, A. Evaluation of some sources of variability in using small mammals as pollution biomonitors. *Chemosphere*, **71**, 2060–2067 (2008).

Milton, A., Cooke, J. A. & Johnson, M. S. Accumulation of Lead, Zinc, and Cadmium in a Wild Population of Clethrionomys glareolus from an Abandoned Lead Mine. *Archives of Environmental Contamination and Toxicology*,**44**, 0405–0411 (2003).

Peig, J. & Green, A. J. The paradigm of body condition: a critical reappraisal of current methods based on mass and length. *Functional Ecology*, **24**, 1323–1332 (2010).

Supplementary Table S 11 – **Quality control of wood mouse analyses for bovine liver (BCR-185R), peach leaves (NIST 1547), dogfish liver (DOLT 4), and fish protein (DORM 3).**

	Pb<br>ICP-MS	Cd<br>ICP-AES	Cu<br>ICP-AES	Zn<br>ICP-AES
LOD ($\mu g \cdot kg^{-1}$)	0.09	0.04	0.5	0.2
BCR-185R ($mg \cdot kg^{-1}$)				
a	0.18	0.54	290	141
b	0.18	0.54	282	140
c	0.19	0.57	290	146
d	0.20	0.57	282	125
e	0.17	0.57	278	124
f	0.30	0.58	280	123
Certified	*0.172*	*0.544*	*277*	*138.6*
NIST 1547 ($mg \cdot kg^{-1}$)				
a	0.63	**0.013**	4.1	20.1
b	-	0.027	3.9	16.8
Certified	*0.87*	*0.026*	*3.7*	*17.9*
DOLT 4 ($mg \cdot kg^{-1}$)				
a	0.13	28.8	36.8	121
Certified	*0.16*	*24.3*	*31.2*	*116*
DORM 3 ($mg \cdot kg^{-1}$)				
a	-	0.34	16.8	53.6
Certified		*0.29*	*15.5*	*51.3*

Supplementary Table S 12 – Results of TMs in wood mouse kidneys.*ICP-MS

ID	region	plot	trap	sex	age	mass	size	liver	kidneys	Cd	Cu	Pb*	Zn
M4	Morvan	M0-11	INRA	F	A	16.80	83.99	1.02	0.22	1.64	18.22	0.29	76.88
M6	Morvan	M0-11	INRA	F	J	11.31	73.55	0.55	0.17	0.91	20.39	0.17	99.39
M16	Morvan	M0-14	SNAP	M	A	31.34	100.51	1.55	0.36	0.97	18.37	0.23	81.00
M17	Morvan	M0-14	INRA	F	J	13.84	85.66	0.67	0.19	1.05	20.36	0.44	99.54
M21	Morvan	M0-15	INRA	F	J	12.80	75.80	0.65	0.20	1.13	19.86	0.99	146.10
M23	Morvan	M0-15	INRA	M	SA	19.13	87.88	1.14	0.23	1.00	16.43	1.24	68.20
M33	Morvan	M0-15	INRA	F	SA	17.61	88.37	0.90	0.18	0.60	21.01	1.71	174.48
M41	Morvan	M0-34	INRA	F	SA	17.44	83.35	0.78	0.19	1.95	19.15	0.45	122.80
M42	Morvan	M0-34	INRA	M	A	35.70	100.92	0.53	0.20	0.90	20.75	0.68	172.67
M44	Morvan	M0-34	INRA	M	J	12.78	76.20	0.58	0.17	0.34	21.40	0.30	118.11
M51	Morvan	M0-44	INRA	F	SA	17.07	81.70	0.93	0.21	3.21	18.17	0.32	148.52
M57	Morvan	M0-44	INRA	F	SA	17.04	80.06	1.05	0.21	2.51	18.71	0.42	74.42
M66	Morvan	M0-55	INRA	M	SA	21.12	86.42	1.28	0.30	1.43	16.92	0.72	92.68
M67	Morvan	M0-55	SNAP	M	A	27.01	88.53	1.29	0.32	4.64	17.65	0.24	72.84
M68	Morvan	M0-55	INRA	F	A	28.24	105.66	1.59	0.35	1.15	11.36	0.82	64.19
M71	Morvan	M0-55	SNAP	M	A	24.66	95.64	1.33	0.28	4.46	16.91	0.11	74.65
M92 #1	Morvan	M0-64	INRA	M	SA	18.88	84.99	1.33	0.24	1.34	15.99	0.53	67.01
M92 #2	Morvan									1.19	15.02	0.213	66.31
M95	Morvan	M0-64	SNAP	F	A	19.84	89.56	1.11	0.27	0.70	16.18	0.26	73.62
M96	Morvan	M0-64	INRA	M	J	16.97	77.60	1.34	0.24	1.05	17.23	0.15	79.53
M102	Morvan	M0-64	INRA	F	A	26.74	86.93	1.41	0.37	1.89	19.24	0.59	94.22
M106	Morvan	M0-64	INRA	F	J	15.11	84.94	0.87	0.22	1.93	17.49	0.43	115.27
M111	Morvan	M0-78	INRA	F	A	24.82	88.87	1.34	0.32	2.42	15.26	0.51	69.67
M114	Morvan	M0-78	SNAP	M	A	24.20	101.28	0.90	0.30	3.07	20.07	0.21	95.13
M116	Morvan	M0-78	INRA	M	A	24.82	97.06	1.47	0.34	1.61	10.37	0.21	44.49
M119	Morvan	M0-78	INRA	F	SA	18.59	76.41	0.97	0.24	1.37	17.81	0.24	79.60
M122	Morvan	M0-88	INRA	M	J	16.86	85.45	0.89	0.23	0.87	17.20	0.23	78.79
M123	Morvan	M0-88	INRA	M	SA	19.49	86.09	1.16	0.26	2.04	18.63	0.21	86.50
M138	Morvan	M0-88	INRA	M	SA	20.07	74.99	1.08	0.21	1.36	17.59	0.39	90.89
M142	Morvan	M0-88	INRA	M	SA	17.65	87.89	1.13	0.25	1.71	16.73	0.30	153.00
M146	Morvan	M0-88	INRA	F	A	24.36	91.60	1.53	0.37	1.28	14.76	0.14	74.27
M421	Morvan	M1-19	INRA	F	A	16.58	80.48	0.65	0.17	2.37	21.28	1.21	86.98
M422	Morvan	M1-19	INRA	F	A	18.68	82.07	0.91	0.21	2.29	17.42	0.67	71.54
M423	Morvan	M1-21	SNAP	M	A	22.46	90.68	1.01	0.20	7.05	18.71	0.05	80.87
M424	Morvan	M1-21	SNAP	F	SA	20.27	83.61	0.88	0.19	2.21	19.92	0.07	75.72
M425	Morvan	M1-21	INRA	F	A	17.62	90.86	0.90	0.20	5.10	18.39	1.62	133.79
M430	Morvan	M1-31	INRA	M	SA	20.19	82.45	1.04	0.23	1.74	11.85	0.35	61.30
M432	Morvan	M1-31	INRA	F	SA	18.17	81.87	1.03	0.32	1.06	14.09	0.18	72.06
M433	Morvan	M1-31	INRA	F	A	24.80	95.72	1.34	0.33	2.55	14.91	0.78	71.35
M435	Morvan	M1-38	INRA	F	SA	18.99	86.54	1.04	0.24	1.51	16.66	2.53	76.37
M437	Morvan	M1-42	INRA	F	SA	18.77	89.82	1.36	0.24	5.61	15.31	0.63	76.27
M438	Morvan	M1-42	INRA	F	SA	20.90	95.33	0.94	0.25	1.62	17.20	0.45	64.21
M442	Morvan	M1-42	INRA	M	A	22.99	77.89	1.52	0.30	3.42	14.88	0.23	69.52
M443	Morvan	M1-42	SNAP	M	SA	17.78	88.93	0.80	0.21	3.48	17.56	0.37	80.77
M444	Morvan	M1-42	SNAP	F	A	22.25	93.03	0.99	0.23	2.68	15.75	0.77	76.55
M445	Morvan	M1-42	INRA	M	SA	19.97	87.65	0.96	0.24	4.41	18.07	0.54	77.94
M447	Morvan	M1-70	INRA	F	J	15.67	79.50	0.80	0.22	0.55	15.90	0.29	72.26
M466	Morvan	M1-80	INRA	F	A	22.45	84.69	0.84	0.24	2.81	17.16	0.23	78.89
M473	Morvan	M1-80	SNAP	F	A	26.79	97.31	1.37	0.32	8.00	14.61	0.14	64.77
M482	Morvan	M1-80	INRA	M	J	14.43	84.37	0.65	0.18	1.30	16.83	0.88	91.78
M483	Morvan	M1-80	INRA	F	A	23.27	94.30	1.22	0.30	4.00	15.21	0.24	73.10
M484	Morvan	M1-80	SNAP	F	A	19.41	85.80	1.05	0.24	3.17	16.31	0.14	69.10
M486	Morvan	M1-82	SNAP	F	SA	20.19	82.45	0.79	0.21	3.39	15.48	0.54	74.27
M489	Morvan	M1-82	INRA	F	SA	19.17	86.01	0.89	0.22	2.64	15.06	1.15	67.72
M490	Morvan	M1-82	INRA	F	SA	17.54	85.11	0.77	0.24	2.20	16.67	2.17	105.44
M491	Morvan	M1-82	SNAP	M	J	16.53	82.79	0.85	0.20	5.26	19.08	1.18	77.20
M492	Morvan	M1-82	SNAP	M	SA	20.07	95.11	0.89	0.23	4.78	17.85	0.44	71.17
M497	Morvan	M1-89	SNAP	F	A	24.69	94.46	1.12	0.30	8.93	14.36	0.32	69.20
M501	Morvan	M1-89	SNAP	M	SA	20.43	93.43	1.08	0.21	1.94	15.44	0.74	65.44
M507 #1	Morvan	M1-89	INRA	M	SA	17.38	87.52	0.95	0.26	1.36	16.54	0.26	77.94
M507 #2	Morvan									1.08	14.78	0.208	81.81
M508	Morvan	M1-89	INRA	F	J	15.88	85.52	0.91	0.20	2.79	16.18	1.14	132.26
M510	Morvan	M1-89	INRA	M	SA	17.38	84.93	0.80	0.26	2.91	18.54	1.37	132.45
M178	Morvan	M2-23	INRA	F	A	23.60	99.02	1.46	0.29	26.83	12.36	1.39	88.02
M179	Morvan	M2-23	SNAP	F	A	22.25	93.74	0.99	0.23	6.58	14.00	0.83	69.62
M180	Morvan	M2-23	SNAP	M	A	25.42	99.27	1.14	0.29	5.19	18.99	2.22	79.69
M182	Morvan	M2-23	SNAP	M	SA	18.00	84.06	0.82	0.17	1.40	21.61	0.75	86.26
M183	Morvan	M2-23	INRA	M	A	23.58	95.13	1.49	0.28	2.53	16.80	1.61	78.92
M187	Morvan	M2-23	INRA	F	J	15.30	89.55	0.66	0.18	3.11	16.99	2.42	115.67
M195	Morvan	M2-27	INRA	M	A	27.32	100.29	1.16	0.28	12.36	18.19	5.02	145.44
M202	Morvan	M2-27	SNAP	F	A	22.82	87.35	1.11	0.33	3.67	17.81	2.09	87.49
M205	Morvan	M2-27	INRA	M	SA	20.70	78.44	0.93	0.37	4.71	18.61	0.77	125.84
M207	Morvan	M2-32	INRA	M	SA	20.51	83.82	0.61	0.15	0.98	19.62	1.81	88.32
M208	Morvan	M2-32	SNAP	M	J	15.73	91.97	0.84	0.26	1.97	20.14	1.24	84.81
M210	Morvan	M2-32	SNAP	F	A	24.30	97.67	1.33	0.27	14.02	17.67	1.07	88.40
M212	Morvan	M2-32	SNAP	M	A	23.48	94.54	1.15	0.27	2.61	15.84	0.98	71.97
M217	Morvan	M2-32	SNAP	F	SA	19.80	85.43	0.91	0.26	2.52	17.58	1.26	81.14
M220	Morvan	M2-32	INRA	F	J	13.58	81.18	0.73	0.19	0.50	14.91	1.55	75.11
M227	Morvan	M2-39	SNAP	F	A	18.84	91.01	0.86	0.24	4.58	17.03	1.33	76.92
M228	Morvan	M2-39	SNAP	F	SA	18.46	88.89	0.84	0.24	3.14	15.00	0.52	80.39
M229	Morvan	M2-39	SNAP	M	SA	20.48	90.15	0.94	0.24	7.61	16.49	0.73	71.63
M232 #1	Morvan	M2-39	SNAP	F	A	22.87	92.52	1.09	0.29	14.43	16.29	0.56	74.82
M232 #2	Morvan									14.72	16.33	-	70.30
M233	Morvan	M2-39	INRA	M	A	24.98	99.84	1.07	0.27	9.17	18.26	1.77	119.25
M236	Morvan	M2-39	SNAP	F	SA	18.30	92.08	0.86	0.26	14.16	16.60	1.54	145.69
M250	Morvan	M2-45	INRA	M	A	24.57	101.05	1.16	1.77	2.15	18.90	1.83	131.62
M255	Morvan	M2-45	SNAP	M	SA	18.39	92.52	0.89	0.26	0.81	18.57	0.59	78.25
M258	Morvan	M2-45	INRA	M	SA	20.09	83.63	0.99	0.31	10.17	17.21	0.51	158.98
M262	Morvan	M2-45	INRA	F	SA	19.02	92.64	0.91	0.25	1.41	17.80	1.19	143.09
M264 #1	Morvan	M2-75	INRA	M	SA	21.81	91.11	1.38	0.25	2.62	17.30	0.79	74.17
M264 #2	Morvan									2.38	16.01	1.230	71.67
M266	Morvan	M2-75	INRA	F	SA	20.76	86.76	1.30	0.26	2.77	16.05	1.91	74.64
M273	Morvan	M2-75	INRA	M	SA	19.09	91.35	1.07	0.23	0.30	13.92	0.38	64.89
M289	Morvan	M2-82	INRA	M	J	13.36	84.91	0.54	0.21	0.17	18.49	0.66	168.04
M290	Morvan	M2-91	INRA	F	A	23.84	88.04	1.32	0.31	0.41	15.38	0.15	66.44
M336	Cévennes	C0-1	INRA	F	A	17.24	77.92	1.17	0.33	0.42	18.53	0.40	81.68
M337	Cévennes	C0-1	SNAP	F	J	9.11	68.10	0.50	0.15	0.05	19.28	0.38	83.44
M338	Cévennes	C0-3	INRA	F	J	12.26	74.28	0.74	0.18	0.21	20.66	0.38	104.88
M339	Cévennes	C0-3	INRA	F	J	12.45	76.06	0.74	0.22	0.22	19.58	0.59	100.70
M342	Cévennes	C0-26	INRA	M	J	15.62	90.99	0.92	0.24	0.10	17.03	0.79	71.22
M346	Cévennes	C0-24	SNAP	F	J	14.36	84.20	0.79	0.23	0.23	16.43	0.13	74.93
M347	Cévennes	C0-24	INRA	M	J	14.31	87.58	0.64	0.25	0.14	21.18	0.31	124.63
M349	Cévennes	C0-48	INRA	M	J	13.75	78.30	0.86	0.24	0.29	18.32	0.30	74.20
M352	Cévennes	C0-51	INRA	M	A	26.80	93.80	1.80	0.47	1.78	17.10	0.36	70.89
M354	Cévennes	C0-51	SNAP	F	J	12.71	80.05	0.71	0.24	0.33	19.76	0.48	72.63
M355	Cévennes	C0-51	INRA	F	SA	16.44	77.74	1.08	0.26	0.64	17.74	0.28	68.11
M357	Cévennes	C0-51	INRA	F	A	21.52	97.49	1.36	0.27	0.68	18.79	0.90	105.87
M358	Cévennes	C0-59	SNAP	M	SA	18.39	90.69	0.89	0.33	0.69	16.50	0.83	64.66
M362	Cévennes	C0-59	SNAP	M	A	23.04	97.60	0.92	0.33	0.28	18.68	0.24	78.28
M365	Cévennes	C0-70	INRA	M	J	16.68	84.89	1.09	0.24	0.45	17.80	0.15	68.55
M367	Cévennes	C0-70	INRA	M	A	24.18	97.38	1.40	0.28	1.84	18.08	0.43	96.79
M371	Cévennes	C0-76	INRA	F	SA	20.62	85.74	1.38	0.35	0.42	15.22	0.29	63.48
M372	Cévennes	C0-76	SNAP	F	A	15.64	86.12	0.95	0.20	0.31	19.77	0.24	72.89
M376	Cévennes	C1-3	INRA	M	A	23.43	96.60	1.13	0.31	0.18	17.51	1.48	82.84
M377	Cévennes	C1-3	INRA	M	A	35.10	95.32	2.21	0.52	2.35	14.30	0.22	55.67
M385	Cévennes	C1-3	INRA	F	J	14.64	77.13	0.73	0.22	0.11	16.48	0.27	76.36
M387	Cévennes	C1-12	SNAP	M	A	30.83	101.81	1.38	0.30	1.15	17.66	0.08	69.61
M388	Cévennes	C1-12	INRA	M	A	27.55	89.53	1.51	0.36	1.37	16.65	0.24	74.53
M395	Cévennes	C1-12	SNAP	F	A	29.41	101.08	1.77	0.33	3.20	15.55	0.21	65.76
M390	Cévennes	C1-86	SNAP	F	A	32.23	96.94	2.14	0.50	3.38	16.38	0.32	68.70
M392 #1	Cévennes	C1-86	SNAP	M	A	25.80	95.15	1.36	0.30	5.98	15.18	0.17	73.30
M392 #2	Cévennes									5.78	15.53	0.202	73.98
M396	Cévennes	C1-96	SNAP	M	SA	21.28	88.47	0.79	0.22	1.60	20.59	0.12	83.36
M397	Cévennes	C1-96	INRA	F	A	25.13	90.70	1.14	0.31	19.59	17.85	0.80	150.23
M399	Cévennes	C1-96	SNAP	M	SA	20.96	84.12	0.71	0.23	0.32	18.51	0.10	75.33
M402	Cévennes	C1-55	INRA	M	J	13.01	75.57	0.63	0.21	0.26	16.30	0.10	70.26
M405	Cévennes	C1-56	INRA	F	J	15.85	77.08	1.04	0.29	0.16	18.63	0.07	71.08
M406	Cévennes	C1-57	INRA	M	A	25.48	84.06	1.39	0.33	4.79	15.17	0.33	72.81
M410	Cévennes	C1-65	INRA	F	SA	18.28	91.70	0.89	0.25	38.08	17.95	3.62	156.98
M413	Cévennes	C1-65	INRA	M	SA	19.24	95.22	0.97	0.29	3.44	19.08	1.36	89.92
M415	Cévennes	C1-65	SNAP	F	J	8.15	67.79	0.35	0.12	1.12	14.39	3.25	85.09
M417	Cévennes	C1-65	INRA	M	A	29.52	96.12	1.63	0.31	13.58	17.08	0.25	88.00
M291	Cévennes	C2-31	INRA	M	A	23.40	92.71	1.18	0.28	0.38	15.07	4.40	64.47
M292	Cévennes	C2-31	SNAP	M	SA	17.62	84.47	1.19	0.25	0.13	18.20	1.45	83.33
M294	Cévennes	C2-31	INRA	M	J	14.99	90.17	0.94	0.21	0.18	22.22	2.39	88.36
M295	Cévennes	C2-31	SNAP	M	SA	18.71	86.95	1.17	0.25	0.27	18.08	0.59	75.72
M297	Cévennes	C2-31	INRA	M	SA	17.90	91.68	1.36	0.32	0.59	17.85	0.31	85.80
M298	Cévennes	C2-31	INRA	F	J	15.03	76.40	1.05	0.21	0.07	20.78	1.41	96.31
M299	Cévennes	C2-31	SNAP	M	SA	17.84	85.34	1.46	0.25	0.08	19.47	2.21	75.95
M300 #1	Cévennes	C2-31	INRA	F	A	31.89	97.16	2.15	0.50	0.58	14.68	4.42	61.38
M300 #2	Cévennes									0.68	15.74	-	61.62
M301	Cévennes	C2-31	SNAP	F	SA	16.15	76.29	1.18	0.23	0.11	17.17	1.72	73.16
M302	Cévennes	C2-31	INRA	F	J	15.87	80.67	1.19	0.22	0.27	17.01	4.01	82.14
M303	Cévennes	C2-31	INRA	M	SA	21.86	94.69	1.34	0.28	0.30	18.50	0.62	71.36
M305	Cévennes	C2-48	INRA	M	J	14.78	82.54	1.08	0.19	0.42	19.46	5.87	79.99
M307	Cévennes	C2-49	INRA	F	A	22.24	83.86	1.71	0.35	0.76	18.34	3.56	77.35
M314 #1	Cévennes	C2-61	INRA	F	A	19.14	96.31	1.03	0.36	1.81	18.41	0.99	118.91
M314 #2	Cévennes									1.82	18.51	1.070	117.82
M315	Cévennes	C2-68	INRA	M	SA	17.81	91.83	1.27	0.29	0.45	19.60	19.40	75.20
M316	Cévennes	C2-68	INRA	F	SA	16.61	81.26	1.15	0.27	0.10	15.99	4.60	61.52
M317	Cévennes	C2-68	INRA	F	J	12.84	75.43	0.78	0.18	0.11	20.56	0.96	77.21
M318	Cévennes	C2-68	SNAP	F	A	23.81	93.41	1.30	0.42	0.80	19.22	2.62	66.78
M319	Cévennes	C2-68	SNAP	M	J	15.76	77.04	0.66	0.20	0.09	20.59	0.76	144.61
M320	Cévennes	C2-68	INRA	M	SA	20.39	87.99	1.19	0.26	0.13	17.65	0.24	70.50
M322	Cévennes	C2-75	SNAP	F	A	22.80	88.87	1.11	0.33	0.31	17.69	0.35	89.24
M324	Cévennes	C2-75	INRA	F	A	27.70	91.46	1.82	0.40	4.11	16.10	0.43	99.78
M325	Cévennes	C2-75	INRA	M	J	13.64	80.54	0.84	0.18	0.08	20.44	1.36	122.48
M327	Cévennes	C2-75	SNAP	M	J	11.60	72.25	0.76	0.19	0.14	20.08	0.87	89.54
M328	Cévennes	C2-78	INRA	M	A	24.72	94.87	1.63	0.38	1.36	18.62	2.52	84.89
M329	Cévennes	C2-78	SNAP	F	A	31.82	99.38	2.15	0.50	3.16	16.08	9.35	63.75
M330	Cévennes	C2-78	INRA	M	J	12.46	72.65	0.75	0.20	0.08	18.77	0.56	139.30
M331	Cévennes	C2-78	INRA	M	A	30.31	92.88	1.58	0.47	0.34	15.70	0.24	59.84
M332	Cévennes	C2-89	INRA	F	J	15.09	86.20	0.16	0.86	0.14	18.54	0.99	159.93
M334	Cévennes	C2-89	INRA	F	SA	16.48	86.91	1.02	0.24	0.13	18.68	0.73	119.36

Supplementary Table S 13 – **R results of backward regression for TMs in wood mouse kidneys.**

Reading the data

ETMs in soils

```
a =read.table(file="sol_total.csv",sep=";",dec=".",header=T)
a$Secteur = factor(a$Secteur,c("GI","RU","CH","VE","RA","SC"),
                   labels=c("M0","M1","M2","C0","C1","C2"))
```

ETMs in wood mice

```
b=read.csv("mulot_total.csv",sep=";", dec=".",header=TRUE)
b$secteur = factor(b$secteur,c("GI","RU","CH","VE","RA","SC"),
                   labels=c("M0","M1","M2","C0","C1","C2"))
b$age<-factor(b$age,c("J","SA","A"))
```

Model selection using backward regression for trace metals in wood mouse kidneys

Cu

```
ModCu <- lm(log10(Cu)~ secteur+log10(Cu_pond)+sexe+mass+
              log10(Cu_pond):sexe+sexe:mass, data=b)
drop1(ModCu, test="F")

## Single term deletions
##
## Model:
## log10(Cu) ~ secteur + log10(Cu_pond) + sexe + mass + log10(Cu_pond):sexe +
##     sexe:mass
##                     Df Sum of Sq     RSS     AIC F value Pr(>F)
## <none>                           0.33624 -942.95
## secteur              5 0.0206097 0.35685 -943.61  1.7898 0.1184
## log10(Cu_pond):sexe  1 0.0058125 0.34205 -942.26  2.5239 0.1143
## sexe:mass            1 0.0032973 0.33953 -943.42  1.4317 0.2334

drop1(update(ModCu, ~ . -sexe:mass), test = "F")

## Single term deletions
##
## Model:
## log10(Cu) ~ secteur + log10(Cu_pond) + sexe + mass + log10(Cu_pond):sexe
##                     Df Sum of Sq     RSS     AIC F value    Pr(>F)
## <none>                           0.33953 -943.42
## secteur              5  0.021325 0.36086 -943.86  1.8465    0.1073
## mass                 1  0.043881 0.38341 -926.34 18.9983 2.445e-05 ***
## log10(Cu_pond):sexe  1  0.005649 0.34518 -942.83  2.4458    0.1200
## ---
## Signif. codes:  0 '***' 0.001 '**' 0.01 '*' 0.05 '.' 0.1 ' ' 1

drop1(update(ModCu, ~ . -sexe:mass -log10(Cu_pond):sexe), test = "F")
```

```
## Single term deletions
##
## Model:
## log10(Cu) ~ secteur + log10(Cu_pond) + sexe + mass
##                  Df Sum of Sq     RSS     AIC F value    Pr(>F)
## <none>                        0.34518 -942.83
## secteur           5  0.023632 0.36881 -942.43  2.0265   0.07814 .
## log10(Cu_pond)    1  0.002880 0.34806 -943.52  1.2348   0.26828
## sexe              1  0.012024 0.35721 -939.45  5.1552   0.02462 *
## mass              1  0.043338 0.38852 -926.26 18.5817 2.956e-05 ***
## ---
## Signif. codes:  0 '***' 0.001 '**' 0.01 '*' 0.05 '.' 0.1 ' ' 1
```

```
drop1(update(ModCu, ~ . -sexe:mass -log10(Cu_pond):sexe -log10(Cu_pond)),
      test = "F")
```

```
## Single term deletions
##
## Model:
## log10(Cu) ~ secteur + sexe + mass
##         Df Sum of Sq     RSS     AIC F value   Pr(>F)
## <none>                0.34806 -943.52
## secteur  5  0.029227 0.37729 -940.87  2.5023  0.03300 *
## sexe     1  0.012241 0.36030 -940.10  5.2401  0.02348 *
## mass     1  0.049127 0.39719 -924.80 21.0304 9.51e-06 ***
## ---
## Signif. codes:  0 '***' 0.001 '**' 0.01 '*' 0.05 '.' 0.1 ' ' 1
```

Zn

```
ModZn <- lm(log10(Zn)~ secteur+log10(Zn_pond)+sexe+mass+
                      log10(Zn_pond):sexe+sexe:mass, data=b)
drop1(ModZn, test="F")
```

```
## Single term deletions
##
## Model:
## log10(Zn) ~ secteur + log10(Zn_pond) + sexe + mass + log10(Zn_pond):sexe +
##     sexe:mass
##                     Df Sum of Sq    RSS     AIC F value  Pr(>F)
## <none>                           1.7494 -684.02
## secteur              5  0.142727 1.8921 -681.71  2.3823 0.04123 *
## log10(Zn_pond):sexe  1  0.013247 1.7627 -684.84  1.1056 0.29478
## sexe:mass            1  0.006948 1.7564 -685.40  0.5798 0.44761
## ---
## Signif. codes:  0 '***' 0.001 '**' 0.01 '*' 0.05 '.' 0.1 ' ' 1
```

```
drop1(update(ModZn, ~ . -sexe:mass), test = "F")
```

```
## Single term deletions
##
## Model:
## log10(Zn) ~ secteur + log10(Zn_pond) + sexe + mass + log10(Zn_pond):sexe
##                     Df Sum of Sq    RSS     AIC F value    Pr(>F)
## <none>                           1.7564 -685.40
```

```
## secteur               5  0.143279 1.8996 -683.09  2.3984 0.0400017 *
## mass                  1  0.150590 1.9069 -674.49 12.6038 0.0005175 ***
## log10(Zn_pond):sexe   1  0.015221 1.7716 -686.05  1.2739 0.2608688
## ---
## Signif. codes:  0 '***' 0.001 '**' 0.01 '*' 0.05 '.' 0.1 ' ' 1
```

```
drop1(update(ModZn, ~ . -sexe:mass -log10(Zn_pond):sexe), test = "F")
```

```
## Single term deletions
##
## Model:
## log10(Zn) ~ secteur + log10(Zn_pond) + sexe + mass
##                Df Sum of Sq    RSS     AIC F value  Pr(>F)
## <none>                      1.7716 -686.05
## secteur         5  0.148108 1.9197 -683.44  2.4746 0.03476 *
## log10(Zn_pond)  1  0.033174 1.8048 -685.13  2.7714 0.09808 .
## sexe            1  0.000264 1.7718 -688.02  0.0221 0.88207
## mass            1  0.148473 1.9201 -675.41 12.4037 0.00057 ***
## ---
## Signif. codes:  0 '***' 0.001 '**' 0.01 '*' 0.05 '.' 0.1 ' ' 1
```

```
drop1(update(ModZn, ~ . -sexe:mass -log10(Zn_pond):sexe -sexe), test = "F")
```

```
## Single term deletions
##
## Model:
## log10(Zn) ~ secteur + log10(Zn_pond) + mass
##                Df Sum of Sq    RSS     AIC F value   Pr(>F)
## <none>                      1.7718 -688.02
## secteur         5  0.148003 1.9198 -685.43  2.4892 0.033807 *
## log10(Zn_pond)  1  0.033248 1.8051 -687.11  2.7959 0.096605 .
## mass            1  0.151751 1.9236 -677.12 12.7612 0.000477 ***
## ---
## Signif. codes:  0 '***' 0.001 '**' 0.01 '*' 0.05 '.' 0.1 ' ' 1
```

```
drop1(update(ModZn, ~ . -sexe:mass -log10(Zn_pond):sexe -sexe -log10(Zn_pond)),
      test = "F")
```

```
## Single term deletions
##
## Model:
## log10(Zn) ~ secteur + mass
##         Df Sum of Sq    RSS     AIC F value    Pr(>F)
## <none>               1.8051 -687.11
## secteur  5   0.15401 1.9591 -684.25  2.5597 0.0296643 *
## mass     1   0.16762 1.9727 -675.16 13.9288 0.0002689 ***
## ---
## Signif. codes:  0 '***' 0.001 '**' 0.01 '*' 0.05 '.' 0.1 ' ' 1
```

Pb

```
ModPb <- lm(log10(Pb)~ secteur+log10(Pb_pond)+sexe+mass+
                    log10(Pb_pond):sexe+sexe:mass, data=b)
drop1(ModPb, test="F")
```

```
## Single term deletions
```

```
##
## Model:
## log10(Pb) ~ secteur + log10(Pb_pond) + sexe + mass + log10(Pb_pond):sexe +
##     sexe:mass
##                     Df Sum of Sq    RSS     AIC F value    Pr(>F)
## <none>                           20.542 -297.30
## secteur              5    7.0913 27.634 -260.74 10.0799 2.631e-08 ***
## log10(Pb_pond):sexe  1    0.0194 20.562 -299.15  0.1382    0.7107
## sexe:mass            1    0.0012 20.544 -299.29  0.0082    0.9280
## ---
## Signif. codes:  0 '***' 0.001 '**' 0.01 '*' 0.05 '.' 0.1 ' ' 1
```

```r
drop1(update(ModPb, ~ . -sexe:mass), test = "F")
```

```
## Single term deletions
##
## Model:
## log10(Pb) ~ secteur + log10(Pb_pond) + sexe + mass + log10(Pb_pond):sexe
##                     Df Sum of Sq    RSS     AIC F value    Pr(>F)
## <none>                           20.544 -299.29
## secteur              5    7.2922 27.836 -261.60 10.4359 1.386e-08 ***
## mass                 1    0.0185 20.562 -301.15  0.1326    0.7163
## log10(Pb_pond):sexe  1    0.0194 20.563 -301.14  0.1391    0.7097
## ---
## Signif. codes:  0 '***' 0.001 '**' 0.01 '*' 0.05 '.' 0.1 ' ' 1
```

```r
drop1(update(ModPb, ~ . -sexe:mass -mass), test = "F")
```

```
## Single term deletions
##
## Model:
## log10(Pb) ~ secteur + log10(Pb_pond) + sexe + log10(Pb_pond):sexe
##                     Df Sum of Sq    RSS     AIC F value    Pr(>F)
## <none>                           20.562 -301.15
## secteur              5    7.4201 27.982 -262.77 10.6815 8.871e-09 ***
## log10(Pb_pond):sexe  1    0.0216 20.584 -302.98  0.1555    0.6939
## ---
## Signif. codes:  0 '***' 0.001 '**' 0.01 '*' 0.05 '.' 0.1 ' ' 1
```

```r
drop1(update(ModPb, ~ . -sexe:mass -mass -log10(Pb_pond):sexe), test = "F")
```

```
## Single term deletions
##
## Model:
## log10(Pb) ~ secteur + log10(Pb_pond) + sexe
##                Df Sum of Sq    RSS     AIC F value    Pr(>F)
## <none>                      20.584 -302.98
## secteur         5    7.4179 28.002 -264.67 10.7393 7.872e-09 ***
## log10(Pb_pond)  1    1.3894 21.973 -294.73 10.0576  0.001842 **
## sexe            1    0.4729 21.057 -301.42  3.4234  0.066257 .
## ---
## Signif. codes:  0 '***' 0.001 '**' 0.01 '*' 0.05 '.' 0.1 ' ' 1
```

```r
drop1(update(ModPb, ~ . -sexe:mass -mass -log10(Pb_pond):sexe -sexe), test = "F")
```

```
## Single term deletions
##
```

```
## Model:
## log10(Pb) ~ secteur + log10(Pb_pond)
##                Df Sum of Sq    RSS     AIC F value    Pr(>F)
## <none>                      21.057 -301.42
## secteur         5    7.2858 28.343 -264.77   10.38 1.448e-08 ***
## log10(Pb_pond)  1    1.3097 22.366 -293.94    9.33  0.002668 **
## ---
## Signif. codes:  0 '***' 0.001 '**' 0.01 '*' 0.05 '.' 0.1 ' ' 1
```

Cd

For sites $M0$, $C0$ and $C2$, too many data were below the LOD for Cd concentrations in topsoils. Here we only tested the data for $M1$, $M2$ and $C1$.

```
bCd=b[-which(b$secteur=="M0"),]
bCd=bCd[-which(bCd$secteur=="C0"),]
bCd=bCd[-which(bCd$secteur=="C2"),]
bCd$secteur <- factor(bCd$secteur, levels = c('M1','M2','C1'))

ModCd <- lm(log10(Cd)~ secteur+log10(Cd_pond)+sexe+mass+
              log10(Cd_pond):sexe+sexe:mass, data=bCd)
drop1(ModCd, test="F")

## Single term deletions
##
## Model:
## log10(Cd) ~ secteur + log10(Cd_pond) + sexe + mass + log10(Cd_pond):sexe +
##     sexe:mass
##                     Df Sum of Sq    RSS     AIC F value  Pr(>F)
## <none>                           14.911 -115.72
## secteur              2   1.73918 16.650 -111.00  4.1406 0.01991 *
## log10(Cd_pond):sexe  1   0.06362 14.975 -117.38  0.3029 0.58378
## sexe:mass            1   0.00232 14.914 -117.71  0.0111 0.91654
## ---
## Signif. codes:  0 '***' 0.001 '**' 0.01 '*' 0.05 '.' 0.1 ' ' 1

drop1(update(ModCd, ~ . -sexe:mass), test = "F")

## Single term deletions
##
## Model:
## log10(Cd) ~ secteur + log10(Cd_pond) + sexe + mass + log10(Cd_pond):sexe
##                     Df Sum of Sq    RSS      AIC F value    Pr(>F)
## <none>                           14.914 -117.707
## secteur              2    1.8156 16.729 -112.631  4.3827   0.01599 *
## mass                 1    4.4197 19.333  -99.202 21.3374 1.645e-05 ***
## log10(Cd_pond):sexe  1    0.0634 14.977 -119.372  0.3059   0.58192
## ---
## Signif. codes:  0 '***' 0.001 '**' 0.01 '*' 0.05 '.' 0.1 ' ' 1

drop1(update(ModCd, ~ . -sexe:mass -log10(Cd_pond):sexe), test = "F")

## Single term deletions
##
## Model:
## log10(Cd) ~ secteur + log10(Cd_pond) + sexe + mass
```

```
##                  Df Sum of Sq    RSS     AIC F value    Pr(>F)
## <none>                        14.977 -119.37
## secteur          2    1.7596 16.737 -114.60  4.2884   0.01734 *
## log10(Cd_pond)   1    0.3489 15.326 -119.55  1.7004   0.19633
## sexe             1    0.4537 15.431 -119.01  2.2114   0.14130
## mass             1    4.4031 19.380 -101.01 21.4614 1.539e-05 ***
## ---
## Signif. codes:  0 '***' 0.001 '**' 0.01 '*' 0.05 '.' 0.1 ' ' 1
```

```
drop1(update(ModCd, ~ . -sexe:mass -log10(Cd_pond):sexe -log10(Cd_pond)),
      test = "F")
```

```
## Single term deletions
##
## Model:
## log10(Cd) ~ secteur + sexe + mass
##         Df Sum of Sq    RSS     AIC F value    Pr(>F)
## <none>                15.326 -119.55
## secteur  2    1.5756 16.901 -115.82   3.804   0.02676 *
## sexe     1    0.3999 15.726 -119.52   1.931   0.16881
## mass     1    4.1811 19.507 -102.50  20.188 2.541e-05 ***
## ---
## Signif. codes:  0 '***' 0.001 '**' 0.01 '*' 0.05 '.' 0.1 ' ' 1
```

```
drop1(update(ModCd, ~ . -sexe:mass -log10(Cd_pond):sexe -log10(Cd_pond) -sexe),
      test = "F")
```

```
## Single term deletions
##
## Model:
## log10(Cd) ~ secteur + mass
##         Df Sum of Sq    RSS     AIC F value    Pr(>F)
## <none>                15.726 -119.52
## secteur  2    1.7832 17.509 -115.03  4.2523   0.01781 *
## mass     1    3.9670 19.693 -103.75 18.9196 4.239e-05 ***
## ---
## Signif. codes:  0 '***' 0.001 '**' 0.01 '*' 0.05 '.' 0.1 ' ' 1
```

Supplementary Table S 14 – **R results of backward regression for condition indices in wood mouse kidneys.**

Model selection using backward regression for body condition and somatic indices

Body condition index - BCI (or SMI)

```
modBCI <- lm(smi~secteur+log10(Cd)+log10(Cu)+log10(Pb)+log10(Zn)+sexe+
                    log10(Cd):sexe+log10(Cu):sexe+log10(Pb):sexe+log10(Zn):sexe,
                  data =bBCI)
drop1(modBCI, test="F")

## Single term deletions
##
## Model:
## smi ~ secteur + log10(Cd) + log10(Cu) + log10(Pb) + log10(Zn) +
##     sexe + log10(Cd):sexe + log10(Cu):sexe + log10(Pb):sexe +
##     log10(Zn):sexe
##                Df Sum of Sq    RSS    AIC F value   Pr(>F)
## <none>                      1377.6 367.43
## secteur         5   154.619 1532.2 373.82  3.1203 0.010605 *
## log10(Cd):sexe  1     5.690 1383.3 366.07  0.5742 0.449888
## log10(Cu):sexe  1    75.169 1452.7 373.62  7.5847 0.006674 **
## log10(Pb):sexe  1     7.138 1384.7 366.23  0.7202 0.397532
## log10(Zn):sexe  1   101.426 1479.0 376.37 10.2341 0.001708 **
## ---
## Signif. codes:  0 '***' 0.001 '**' 0.01 '*' 0.05 '.' 0.1 ' ' 1

drop1(update(modBCI, ~ . -log10(Cd):sexe), test = "F")

## Single term deletions
##
## Model:
## smi ~ secteur + log10(Cd) + log10(Cu) + log10(Pb) + log10(Zn) +
##     sexe + log10(Cu):sexe + log10(Pb):sexe + log10(Zn):sexe
##                Df Sum of Sq    RSS    AIC F value    Pr(>F)
## <none>                      1383.3 366.07
## secteur         5   151.567 1534.8 372.08  3.0680 0.0116741 *
## log10(Cd)       1    42.210 1425.5 368.70  4.2721 0.0405849 *
## log10(Cu):sexe  1   104.462 1487.7 375.28 10.5726 0.0014381 **
## log10(Pb):sexe  1     7.401 1390.7 364.89  0.7491 0.3882541
## log10(Zn):sexe  1   119.141 1502.4 376.79 12.0583 0.0006863 ***
## ---
## Signif. codes:  0 '***' 0.001 '**' 0.01 '*' 0.05 '.' 0.1 ' ' 1

drop1(update(modBCI, ~ . -log10(Cd):sexe -log10(Pb):sexe), test = "F")

## Single term deletions
##
## Model:
## smi ~ secteur + log10(Cd) + log10(Cu) + log10(Pb) + log10(Zn) +
##     sexe + log10(Cu):sexe + log10(Zn):sexe
##                Df Sum of Sq    RSS    AIC F value    Pr(>F)
## <none>                      1390.7 364.89
## secteur         5   160.705 1551.4 371.73  3.2588 0.0081474 **
```

```
## log10(Cd)        1    45.188 1435.8 367.81  4.5817 0.0340364 *
## log10(Pb)        1    55.166 1445.8 368.88  5.5933 0.0193912 *
## log10(Cu):sexe  1   106.294 1497.0 374.23 10.7772 0.0012957 **
## log10(Zn):sexe  1   112.927 1503.6 374.91 11.4497 0.0009261 ***
## ---
## Signif. codes:  0 '***' 0.001 '**' 0.01 '*' 0.05 '.' 0.1 ' ' 1
```

Scaled liver index - SLI

```
modSLI <- lm(sli~secteur+log10(Cd)+log10(Cu)+log10(Pb)+log10(Zn)+sexe+
                     log10(Cd):sexe+log10(Cu):sexe+log10(Pb):sexe+log10(Zn):sexe,
                   data=bHSI)
drop1(modSLI, test="F")

## Single term deletions
##
## Model:
## sli ~ secteur + log10(Cd) + log10(Cu) + log10(Pb) + log10(Zn) +
##     sexe + log10(Cd):sexe + log10(Cu):sexe + log10(Pb):sexe +
##     log10(Zn):sexe
##                 Df Sum of Sq     RSS     AIC F value    Pr(>F)
## <none>                        9.8709 -385.61
## secteur          5   2.17373 12.0446 -365.36  6.0339 4.438e-05 ***
## log10(Cd):sexe  1   0.21260 10.0835 -384.37  2.9508   0.08810 .
## log10(Cu):sexe  1   0.36091 10.2318 -382.15  5.0092   0.02683 *
## log10(Pb):sexe  1   0.01932  9.8902 -387.31  0.2681   0.60542
## log10(Zn):sexe  1   0.25340 10.1243 -383.76  3.5170   0.06287 .
## ---
## Signif. codes:  0 '***' 0.001 '**' 0.01 '*' 0.05 '.' 0.1 ' ' 1

drop1(update(modSLI, ~ . -log10(Pb):sexe), test = "F")

## Single term deletions
##
## Model:
## sli ~ secteur + log10(Cd) + log10(Cu) + log10(Pb) + log10(Zn) +
##     sexe + log10(Cd):sexe + log10(Cu):sexe + log10(Zn):sexe
##                 Df Sum of Sq     RSS     AIC F value    Pr(>F)
## <none>                        9.8902 -387.31
## secteur          5   2.22427 12.1145 -366.48  6.2071 3.191e-05 ***
## log10(Pb)        1   0.05605  9.9463 -388.46  0.7821   0.37805
## log10(Cd):sexe  1   0.21591 10.1061 -386.03  3.0127   0.08485 .
## log10(Cu):sexe  1   0.36419 10.2544 -383.82  5.0816   0.02576 *
## log10(Zn):sexe  1   0.23793 10.1282 -385.70  3.3198   0.07061 .
## ---
## Signif. codes:  0 '***' 0.001 '**' 0.01 '*' 0.05 '.' 0.1 ' ' 1

drop1(update(modSLI, ~ . -log10(Pb):sexe -log10(Pb)), test = "F")

## Single term deletions
##
## Model:
## sli ~ secteur + log10(Cd) + log10(Cu) + log10(Zn) + sexe + log10(Cd):sexe +
##     log10(Cu):sexe + log10(Zn):sexe
##                 Df Sum of Sq     RSS     AIC F value   Pr(>F)
```

```
## <none>                           9.9463 -388.46
## secteur          5   2.16837 12.1146 -368.48  6.0606 4.16e-05 ***
## log10(Cd):sexe  1   0.20744 10.1537 -387.32  2.8990  0.09087 .
## log10(Cu):sexe  1   0.39594 10.3422 -384.52  5.5333  0.02006 *
## log10(Zn):sexe  1   0.27151 10.2178 -386.36  3.7943  0.05344 .
## ---
## Signif. codes:  0 '***' 0.001 '**' 0.01 '*' 0.05 '.' 0.1 ' ' 1
```

```
drop1(update(modSLI, ~ . -log10(Pb):sexe -log10(Pb) -log10(Cd):sexe), test = "F")
```

```
## Single term deletions
##
## Model:
## sli ~ secteur + log10(Cd) + log10(Cu) + log10(Zn) + sexe + log10(Cu):sexe +
##     log10(Zn):sexe
##                Df Sum of Sq    RSS     AIC F value    Pr(>F)
## <none>                      10.154 -387.32
## secteur         5   2.08339 12.237 -368.95  5.7452 7.452e-05 ***
## log10(Cd)       1   0.02358 10.177 -388.97  0.3252  0.569434
## log10(Cu):sexe  1   0.74545 10.899 -378.55 10.2783  0.001668 **
## log10(Zn):sexe  1   0.44696 10.601 -382.77  6.1628  0.014229 *
## ---
## Signif. codes:  0 '***' 0.001 '**' 0.01 '*' 0.05 '.' 0.1 ' ' 1
```

```
drop1(update(modSLI, ~ . -log10(Pb):sexe -log10(Pb) -log10(Cd):sexe -log10(Cd)),
      test = "F")
```

```
## Single term deletions
##
## Model:
## sli ~ secteur + log10(Cu) + log10(Zn) + sexe + log10(Cu):sexe +
##     log10(Zn):sexe
##                Df Sum of Sq    RSS     AIC F value    Pr(>F)
## <none>                      10.177 -388.97
## secteur         5   2.62824 12.806 -364.05  7.2825 4.264e-06 ***
## log10(Cu):sexe  1   0.72562 10.903 -380.50 10.0530  0.001866 **
## log10(Zn):sexe  1   0.42457 10.602 -384.75  5.8821  0.016560 *
## ---
## Signif. codes:  0 '***' 0.001 '**' 0.01 '*' 0.05 '.' 0.1 ' ' 1
```

Scaled kidney index

```
modSKI <- lm(ski~secteur+log10(Cd)+log10(Cu)+log10(Pb)+log10(Zn)+sexe+
                    log10(Cd):sexe+log10(Cu):sexe+log10(Pb):sexe+log10(Zn):sexe,
                  data =bRSI)
drop1(modSKI, test="F")
```

```
## Single term deletions
##
## Model:
## ski ~ secteur + log10(Cd) + log10(Cu) + log10(Pb) + log10(Zn) +
##     sexe + log10(Cd):sexe + log10(Cu):sexe + log10(Pb):sexe +
##     log10(Zn):sexe
##                Df Sum of Sq     RSS     AIC F value   Pr(>F)
## <none>                      0.43728 -859.36
```

```
## secteur          5  0.071926 0.50920 -846.22  4.5069 0.000780 ***
## log10(Cd):sexe   1  0.005684 0.44296 -859.40  1.7808 0.184269
## log10(Cu):sexe   1  0.018910 0.45619 -854.93  5.9247 0.016218 *
## log10(Pb):sexe   1  0.000678 0.43795 -861.13  0.2123 0.645729
## log10(Zn):sexe   1  0.022143 0.45942 -853.85  6.9376 0.009411 **
## ---
## Signif. codes:  0 '***' 0.001 '**' 0.01 '*' 0.05 '.' 0.1 ' ' 1
```

```
drop1(update(modSKI, ~ . -log10(Pb):sexe), test = "F")
```

```
## Single term deletions
##
## Model:
## ski ~ secteur + log10(Cd) + log10(Cu) + log10(Pb) + log10(Zn) +
##     sexe + log10(Cd):sexe + log10(Cu):sexe + log10(Zn):sexe
##                 Df Sum of Sq     RSS     AIC F value    Pr(>F)
## <none>                       0.43795 -861.13
## secteur          5  0.074367 0.51232 -847.29  4.6866 0.0005531 ***
## log10(Pb)        1  0.013003 0.45096 -858.68  4.0972 0.0448837 *
## log10(Cd):sexe   1  0.005791 0.44374 -861.13  1.8247 0.1789649
## log10(Cu):sexe   1  0.019076 0.45703 -856.65  6.0108 0.0154679 *
## log10(Zn):sexe   1  0.021489 0.45944 -855.85  6.7713 0.0102750 *
## ---
## Signif. codes:  0 '***' 0.001 '**' 0.01 '*' 0.05 '.' 0.1 ' ' 1
```

```
drop1(update(modSKI, ~ . -log10(Pb):sexe -log10(Cd):sexe), test = "F")
```

```
## Single term deletions
##
## Model:
## ski ~ secteur + log10(Cd) + log10(Cu) + log10(Pb) + log10(Zn) +
##     sexe + log10(Cu):sexe + log10(Zn):sexe
##                 Df Sum of Sq     RSS     AIC F value   Pr(>F)
## <none>                       0.44374 -861.13
## secteur          5  0.072676 0.51642 -848.08  4.5530 0.000709 ***
## log10(Cd)        1  0.001775 0.44552 -862.52  0.5561 0.457089
## log10(Pb)        1  0.012374 0.45612 -858.95  3.8761 0.050968 .
## log10(Cu):sexe   1  0.032344 0.47609 -852.44 10.1317 0.001799 **
## log10(Zn):sexe   1  0.030035 0.47378 -853.18  9.4083 0.002597 **
## ---
## Signif. codes:  0 '***' 0.001 '**' 0.01 '*' 0.05 '.' 0.1 ' ' 1
```

```
drop1(update(modSKI, ~ . -log10(Pb):sexe -log10(Cd):sexe -log10(Cd)), test = "F")
```

```
## Single term deletions
##
## Model:
## ski ~ secteur + log10(Cu) + log10(Pb) + log10(Zn) + sexe + log10(Cu):sexe +
##     log10(Zn):sexe
##                 Df Sum of Sq     RSS     AIC F value    Pr(>F)
## <none>                       0.44552 -862.52
## secteur          5  0.093804 0.53932 -843.48  5.8954 5.625e-05 ***
## log10(Pb)        1  0.011565 0.45708 -860.63  3.6341  0.058658 .
## log10(Cu):sexe   1  0.031188 0.47671 -854.24  9.8004  0.002124 **
## log10(Zn):sexe   1  0.028433 0.47395 -855.12  8.9349  0.003306 **
## ---
## Signif. codes:  0 '***' 0.001 '**' 0.01 '*' 0.05 '.' 0.1 ' ' 1
```

```r
drop1(update(modSKI, ~ . -log10(Pb):sexe -log10(Cd):sexe -log10(Cd) -log10(Pb)),
      test = "F")

## Single term deletions
##
## Model:
## ski ~ secteur + log10(Cu) + log10(Zn) + sexe + log10(Cu):sexe +
##     log10(Zn):sexe
##                Df Sum of Sq     RSS     AIC F value   Pr(>F)
## <none>                      0.45708 -860.63
## secteur         5  0.087213 0.54430 -844.09  5.3807 0.000147 ***
## log10(Cu):sexe  1  0.035499 0.49258 -851.26 10.9506 0.001188 **
## log10(Zn):sexe  1  0.034094 0.49118 -851.69 10.5173 0.001476 **
## ---
## Signif. codes:  0 '***' 0.001 '**' 0.01 '*' 0.05 '.' 0.1 ' ' 1
```

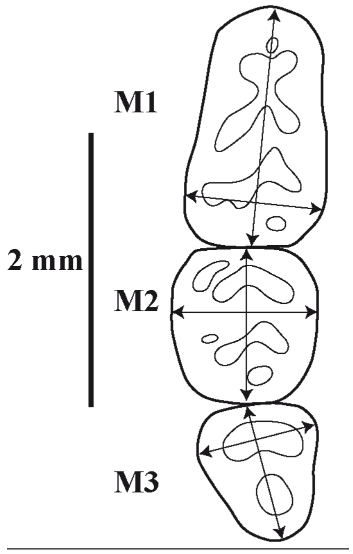

Supplementary Figure S 5 – Bilateral morphological traits selected to study FA in wood mice (*Apodemus sylvaticus*).
M1 stands for the first lower molar, M2 stands for the second lower molar, and M3 stands for the third lower molar.
